# Biological Soil Crust Bacterial Communities Vary Along Climatic and Shrub Cover Gradients Within a Sagebrush Steppe Ecosystem

**DOI:** 10.3389/fmicb.2021.569791

**Published:** 2021-05-06

**Authors:** Yaqi You, Ken Aho, Kathleen A. Lohse, Stacy G. Schwabedissen, Rhesa N. Ledbetter, Timothy S. Magnuson

**Affiliations:** Department of Biological Sciences, Idaho State University, Pocatello, ID, United States

**Keywords:** climate change, nitrogen cycling, cyanobacteria, bacterial community, biological soil crusts, sagebrush steppe, Reynolds Creek Critical Zone Observatory, symbiotic diazotrophs

## Abstract

Numerous studies have examined bacterial communities in biological soil crusts (BSCs) associated with warm arid to semiarid ecosystems. Few, however, have examined bacterial communities in BSCs associated with cold steppe ecosystems, which often span a wide range of climate conditions and are sensitive to trends predicted by relevant climate models. Here, we utilized Illumina sequencing to examine BSC bacterial communities with respect to climatic gradients (elevation), land management practices (grazing vs. non-grazing), and shrub/intershrub patches in a cold sagebrush steppe ecosystem in southwestern Idaho, United States. Particular attention was paid to shifts in bacterial community structure and composition. BSC bacterial communities, including keystone N-fixing taxa, shifted dramatically with both elevation and shrub-canopy microclimates within elevational zones. BSC cover and BSC cyanobacteria abundance were much higher at lower elevation (warmer and drier) sites and in intershrub areas. Shrub-understory BSCs were significantly associated with several non-cyanobacteria diazotrophic genera, including *Mesorhizobium* and *Allorhizobium*-*Neorhizobium*-*Pararhizobium*-*Rhizobium*. High elevation (wetter and colder) sites had distinct, highly diverse, but low-cover BSC communities that were significantly indicated by non-cyanobacterial diazotrophic taxa including families in the order Rhizobiales and the family Frankiaceae. Abiotic soil characteristics, especially pH and ammonium, varied with both elevation and shrub/intershrub level, and were strongly associated with BSC community composition. Functional inference using the PICRUSt pipeline identified shifts in putative N-fixing taxa with respect to both the elevational gradient and the presence/absence of shrub canopy cover. These results add to current understanding of biocrust microbial ecology in cold steppe, serving as a baseline for future mechanistic research.

## Introduction

Drylands are characterized by low precipitation and limited vegetation (Belnap and Lange, 2003; Huang et al., 2016). These ecosystems cover over 35% of terrestrial landmass (Kuske et al., 2012), and are commonly colonized by biological soil crusts (BSCs; Chen et al., 2020). BSCs are a soil-surface community of mosses, lichens, liverworts, algae, archaea, cyanobacteria, and other bacteria. BSCs vary dramatically in morphology from flat to rolling to pinnacled structures, depending on climatic conditions, particularly precipitation and frost-heaving (Belnap, 2003; Belnap and Lange, 2003). BSC communities perform vital ecological functions (Belnap et al., 2016; Bowker et al., 2018), including reducing nutrient loss by runoff (Barger et al., 2006), decreasing erosion by wind (Belnap and Lange, 2003), fixing carbon (C) and nitrogen (N; Belnap, 2003; Belnap and Lange, 2003), facilitating vascular plant establishment (Belnap, 2003; Coe et al., 2014), and decreasing soil surface albedo (Belnap and Lange, 2003; Kuske et al., 2012). Despite their importance in the ecosystem structure and function in drylands, current understanding of how BSC communities respond to changing climatic conditions and environmental disturbances is still incomplete (Belnap, 2002; Belnap and Lange, 2003; Yeager et al., 2004; Kuske et al., 2012; Chen et al., 2020). Addressing these knowledge gaps may be particularly important in environments whose winter precipitation is primarily snow, as these areas are likely to undergo pronounced climate changes, with declines in precipitation amount and shifts in precipitation phase (Nayak et al., 2010; Seyfried et al., 2011; Flerchinger et al., 2019).

Nitrogen often limits primary production in dryland ecosystems (Reed et al., 2011), wherein dinitrogen (N_2_) fixation by BSCs may constitute the largest source of N inputs (Belnap, 2002; Belnap and Lange, 2003; Yeager et al., 2004, 2007; Reed et al., 2011; Barger et al., 2016; Pepe-Ranney et al., 2016; Heindel et al., 2019). In mature BSCs, cyanobacteria are the primary N_2_-fixers as free-living microbes or through associations with lichens and mosses (Belnap, 2002; Muñoz-Martín et al., 2019), whereas non-cyanobacterial heterotrophic diazotrophs, such as *Clostridiaceae* and *Proteobacteria*, mediate N_2_ fixation during the early stages of biocrust development (Barger et al., 2016; Pepe-Ranney et al., 2016). Existing studies have examined the cyanobacterial communities of BSCs from the Colorado Plateau, United States (Yeager et al., 2004, 2007; Gundlapally and Garcia-Pichel, 2006; Kuske et al., 2012), the Chihuahuan Desert, United States (Yeager et al., 2004, 2007), China (Wang et al., 2015), India (Tirkey and Adhikary, 2005; Kumar and Adhikary, 2015), Israel (Kidron et al., 2010; Hagemann et al., 2015), Africa (Dojani et al., 2013), and Australia (Chilton et al., 2018; Moreira-Grez et al., 2019). Across these regions, cyanobacterial taxa typically display greater abundance in well-drained, alkaline pH, saline soils (Belnap and Lange, 2003). In certain BSCs from the southwestern United States, *Microcoleus* spp. are the first to colonize soil, whereas *Nostoc* and *Scytonema* spp. occur in greater abundance as lichens and mosses become established (Belnap, 2002; Yeager et al., 2004; Kuske et al., 2012). In contrast, *Microcoleus* spp. were not detected or much less present in Australia (Chilton et al., 2018; Moreira-Grez et al., 2019). Other taxa that fix N_2_ asymbiotically are from the classes Alphaproteobacteria and Gammaproteobacteria, but their distribution and function in BSCs is not fully understood (Yeager et al., 2007; Barger et al., 2016; Pepe-Ranney et al., 2016). Indeed, investigations of the diversity and compositional patterns of cyanobacteria and other BSC bacterial taxa, vital to N cycling in colder and more mesic drylands, remain very limited (Blay et al., 2017; Steven et al., 2018; Moreira-Grez et al., 2019; Li et al., 2020).

Biological soil crusts in the Intermountain West of the United States generally occur in sagebrush steppe ecosystems with pronounced spatial heterogeneity in soils and other environmental resources, due to patchy distributions of individual shrub canopies (Phillips and MacMahon, 1981). Sagebrush steppe ecosystems are currently experiencing dramatic temperature increases and shifts in precipitation (Nayak et al., 2010; Seyfried et al., 2011). For example, long-term studies at the Reynolds Creek Experimental Watershed (RCEW), a semiarid rangeland watershed representative of vast tracts within the Intermountain West, have documented trends of increasing temperature over the last 50 years, particularly minimum temperature (Nayak et al., 2010). At the RCEW, maximum snow water equivalent and the snow-to-rain ratio have declined at all elevations with the largest and most significant declines at mid and low elevations (Nayak et al., 2010). Shifts in precipitation phase and increased temperatures will likely alter both the distribution and characteristics of sagebrush steppe plant species and communities of BSCs within sagebrush steppe ecosystems (Ferrenberg et al., 2015).

Anthropogenic activities including recreation, invasive species introduction, grazing, fire, and agriculture have directly altered large areas of the Intermountain West with potential concomitant changes to BSC communities (Belnap and Lange, 2003; Barger et al., 2006; Reed et al., 2011; Coe et al., 2012; Kuske et al., 2012). BSCs dominated by lichens and mosses, such as those in the Intermountain West, are considered extremely sensitive to disturbance (Belnap, 2003; Belnap and Lange, 2003), and once disturbed, become dominated by disturbance-tolerant cyanobacteria (Belnap, 2003). Following disturbance, BSC recovery can take over 1,000 years, depending on soil characteristics, severity of disturbance, and climate (Belnap, 2002). Thus, understanding how BSC bacterial communities, particularly taxonomic and functional compositions involved in N_2_ fixation, vary with disturbance and climatic changes is vital to the management of these systems (Belnap et al., 2016; Steven et al., 2018; Chen et al., 2020; Velasco Ayuso et al., 2020).

Several papers have examined variation in BSC taxonomy and biochemistry in cold steppe ecosystems along a well-studied elevational gradient at the RCEW in southwestern Idaho, United States. Schwabedissen et al. (2017) found that BSCs were major contributors of N input to these ecosystems with an average fixation rate of 10–36 kg N/ha/yr based on a conversion factor of 3 moles of C_2_H_2_ reduced:1 mole of N_2_ fixed (e.g., Zaady et al., 1998; Abed et al., 2010; Zhao et al., 2010; Barger et al., 2016), and that nitrogenase activity varied with climatic conditions and shrub-intershrub patches. Specifically, warmer, drier climate at lower elevations was associated with later successional BSC communities (e.g., mosses and lichens) and had higher nitrogenase activity compared to colder, wetter climate at higher elevations. Blay et al. (2017) examined variation in BSC bacterial phyla with respect to elevation at a single time point. These authors found that the abundance of *Actinobacteria* and *Firmicutes* increased whereas *Cyanobacteria* decreased at higher elevations with cooler, wetter climates. In the current study, we significantly extend this prior work by quantifying variation of BSC bacterial communities, with respect to both structure and composition, over multiple seasons and with previously defined environmental factors, namely grazing disturbance, shrub/intershrub patches, and climatic shifts with elevation. Deep sequencing allowed a high-resolution depiction of BSC bacterial communities in cold steppe in the Intermountain West. We had one general hypothesis with specific predictions:

The BSC microbial community, including taxa known to mediate N cycling (e.g., N_2_ fixation), will vary among elevational zones, shrub/intershrub patches, and grazing/exclosure treatments.

- Based on previous work at the study site, we expect that cyanobacteria will be dominant at alkaline, drier, warmer areas, whereas non-cyanobacterial diazotrophs (e.g., rhizobial species) will be more abundant in colder, wetter, acidic areas.
- We expect that bacteria associated with early-successional BSCs, such as the cyanobacteria genus *Microcoleus*, will be more abundant in grazed areas, whereas late-successional cyanobacteria, such as the genus *Nostoc*, will be more abundant in ungrazed areas, as observed in other studies (Belnap, 2002; Yeager et al., 2004; Dojani et al., 2013).
- We expect that higher abundance of cyanobacteria will occur in intershrub areas because BSC cover is typically higher in spaces between shrubs (Belnap, 2003; Elliott et al., 2014). Conversely, symbiotic diazotrophs will be more dominant under shrubs due to their associations with plant roots.

## Materials and Methods

### Study Area

**Figure 1:**
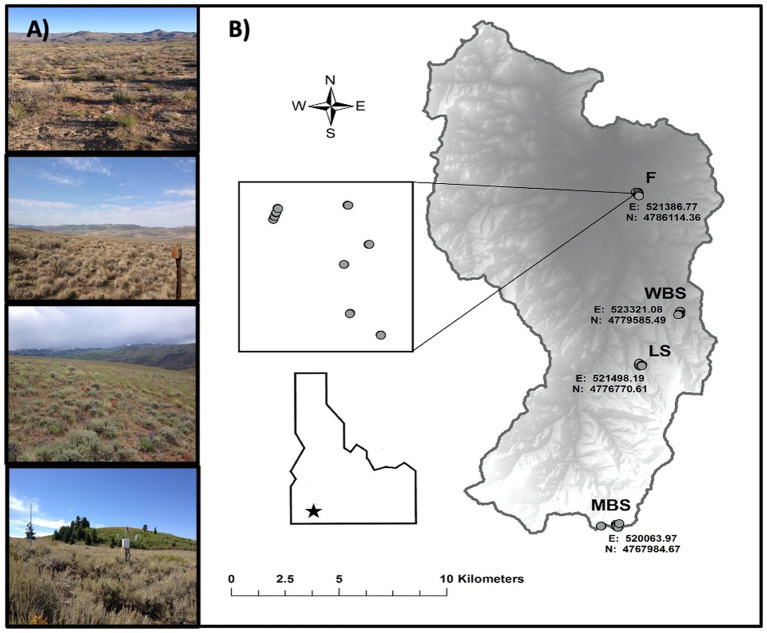
Summary maps for the Reynolds Creek Critical Zone Observatory (RC CZO) in southwest Idaho, United States. **(A)** Photographs of the four main study sites [top to bottom, from lowest to highest elevation, these are F (1,170 m), WBS (1,400 m), LS (1,600 m), and MBS (2080 m)] and sagebrush vegetation. **(B)** Maps showing elevation, and other geographic properties.

Our study was conducted at the RCEW, now the National Science Foundation Reynolds Creek Critical Zone Observatory (RC CZO), located in the Owyhee Range in southwestern Idaho, United States (Figure 1; Seyfried et al., 2018). The Observatory is a 239 km^2^ watershed with a broad elevational range (1170–2080 m). The vegetation of the RC CZO is typical of the Intermountain West with large regions dominated by sagebrush (*Artemisia* sp.), particularly varieties of big sagebrush (*A. tridentata* spp.), quaking aspen forests (*Populus tremuloides*), and juniper woodlands (*Juniperus* sp.). At lower elevations, rain is the predominant form of precipitation, whereas at higher elevations, winter precipitation is primarily snow. Extensive spatial and temporal climate and soil temperature and moisture data have been collected for ≥40 years by the United States Department of Agriculture Agricultural Research Service Northwest Watershed Research Center in Boise, Idaho (Nayak et al., 2010; Seyfried et al., 2011).

### Experimental Design and Sampling

Biocrust samples were collected at four study sites along an elevational gradient (climosequence) within the CZO (Figure 1; Table 1; Schwabedissen et al., 2017). In order of increasing elevation, these sites were designated: Flats = F, Wyoming Big Sage = WBS, Low Sage = LS, and Mountain Big Sage = MBS. These comprised of RC CZO core sites, each equipped with a climate station, soil moisture and temperature probes, and sap flow sensors, and containing grazing exclosures (Figure 1). Three sites (WBS, LS, and MBS) were also equipped with eddy covariance towers. From the lowest to highest elevations, mean annual precipitation (MAP) ranged from 235 to 803 mm, and mean annual temperature (MAT) varied from 9.1 to 5.4°C. Although *A. tridentata* was the dominant shrub species across the elevational gradient, subspecies of *A. tridentata* co-varied with elevation (Table 1). Variation in other soil formation factors (Jenny, 1941) such as parent material, time, and topography was minimized in selection of sample sites. For example, parent material at all sites is volcanic basalt in origin, and topography was relatively flat (<5% slope).

**Table 1:** Description of each sampling site according to their elevation, GPS location (latitude and longitude), mean annual temperature (MAT), mean annual precipitation (MAP), and dominant sagebrush vegetation.

Site	Elevation (m)	Location	MAT (°C)	(mm year^−1^)	Dominant shrub species
F	1,170	43.229 N 116.738 W	9.1	235	*A. tridentata* ssp. *wyomingensis*
WBS	1,400	43.168 N 116.713 W	9.2	298	*A. tridentata* ssp. *wyomingensis*
LS	1,600	43.140 N 116.734 W	8.5	345	*A. arbuscula*
MBS	2,080	43.064 N 116.749 W	5.4	803	*A. tridentata* ssp. *vaseyana*

Sites increasing in elevation are Flats (F), Wyoming Big Sage (WBS), Low Sage (LS), and Mountain Big Sage (MBS). Adapted from Blay et al. (2017) and Schwabedissen et al. (2017).

**Figure 2:**
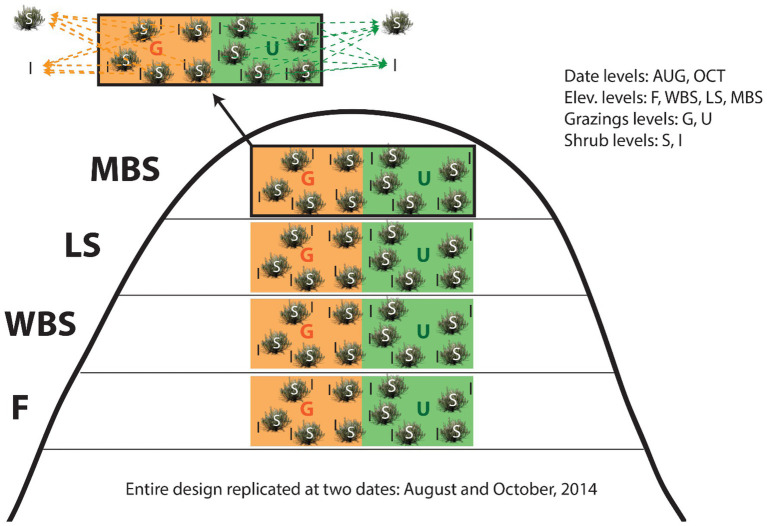
Study experimental design for collecting biological soil crusts (BSCs) and underneath soil samples. Note the split-split plot design structure: shrub levels (split-split plots) within grazing levels (split-plot) within elevation levels (whole-plots) within date blocks (whole plot replicates). G, grazed plots; U, ungrazed plots (exclosure for >40 years); S, shrub plots; I, intershrub plots.

At each elevation, shrub-intershrub biocrust samples were collected on two dates (August and October 2014), from locations associated with five randomly selected shrubs, in both grazed and ungrazed (i.e., exclosure for >40 years) plots (Figure 2). BSC samples were delimited with a 10 × 10 cm metal square, and collected using an ethanol-sterilized spatula and soil knife to a depth of 2.5 cm, the observed maximum thickness of BSCs in this region. After collection, samples were immediately placed in pre-sterilized containers on ice in the field and stored at 4°C in the lab prior to processing.

To increase the capacity for causal inferences, sampling date, elevation, grazing, and shrub/intershrub levels were nested in a split-split plot design, with shrub canopy levels placed in grazing levels, then in elevational levels, and finally in date blocks (Figure 2). Because of resource limitations, shrub/intershrub replicates within grazing levels were amalgamated into single samples for microbial community analyses (Figure 2). Samples were combined by volume rather than DNA mass as had been prior done in Blay et al. (2017) to better estimate true diversity within samples, rather than maximize rare taxonomic outcomes. This study design resulted in 32 independent observations: 2 shrub (shrub/intershrub) observations × 2 grazing (grazed/ungrazed) levels × 4 elevation levels × 2 dates = 32 observations.

For soil biogeochemical analysis, soil cores 5 cm in depth were extracted from under the BSCs sampled in August and October 2014. Soil samples were not amalgamated to create single plant/intershrub observations within grazing levels. Thus, 80 independent soil observations were obtained per date: five samples at each of the 2 shrub levels × 2 grazing (grazed/ungrazed) levels × 4 elevation levels = 80 observations. Temporally dynamic soil variables such as gravimetric water content, nutrient pools as ammonium (NH_4_), nitrate (NO_3_), and orthophosphate (ortho-P), potential net N mineralization, and potential net nitrification were obtained for both dates. Soil organic carbon and nitrogen pools and isotopes of them were obtained for October 2014. Biocrust cover, bare ground cover, and vegetation cover were measured in May 2015.

### Soil Biogeochemistry

Soils were sieved through a 2-mm sieve before determination of pH, electrical conductivity (EC), and nutrient concentrations. Soil pH and EC were determined at a 1:1 ratio of water to soil. Owing to the high organic matter content at the MBS site, a 2:1 ratio was required for assays. We note that although the soil pH was likely unaffected by this 2:1 dilution, the true soil EC value at the MBS site is likely to be twice as high as reported (Schwabedissen et al., 2017). Following the addition of water to soil, samples were stirred for 1 min, and readings were recorded at 1 h using a Dual Channel pH/Ion/Conductivity Meter XL50 (Fisher Scientific, Pittsburg, PA, United States). Soil was extracted for inorganic nutrients, ammonium (NH_4_^+^), and nitrate (NO_3_^−^), using a 1:5 ratio of soil to 2 M potassium chloride (KCl, Fisher Scientific, Pittsburg, PA, United States). Preceding measurement, samples were placed on a shaker for 1 h and extracts were then filtered through pre-KCl leached Whatman #1 filters (Fisher Scientific, Pittsburg, PA, United States). Another set of subsamples was placed in the dark for 7 days under aerobic conditions at either 60% water holding capacity (WHC) or *in situ* moisture conditions to determine potential or *in situ* rates of net N mineralization and nitrification, respectively. Soils were similarly extracted as described above after 7 days. Ortho-phosphate (ortho-P) was extracted using a 1:20 ratio of soil to 0.5 M sodium bicarbonate (NaHCO_3_, Fisher Scientific, Pittsburg, PA, United States). Ortho-P samples were placed on a shaker for 1 h and then passed through Whatman #40 filters (Fisher Scientific, Pittsburg, PA, United States) pre-leached with 0.5 M NaHCO_3_. Samples were stored at 4°C before analyzed on a SmartChem 200 Discrete Analyzer Auto-Spectrophotometer (Westco Scientific Instruments, Inc., Brookfield, CT, United States) for NH_4_^+^ as N with a salicylate method (AMM-003-A) and for NO_3_^−^ as N with a nitrate reduction method with a cadmium metal (NO3-001-A). The ortho-P extracts were analyzed colorimetrically on the SmartChem 200 using the PHO-001-A method. Samples were also analyzed for soil nitrogenase activity as reported in Schwabedissen et al. (2017) and incorporated here as explanatory variables of bacterial community and potential functional analyses.

Carbon and nitrogen mass and *δ*^13^C and δ^15^N isotopic values were determined on an Elemental Combustion System 4,010 (Costech Analytical Tech, Inc., United States) interfaced to a Delta V Advantage Mass Spectrometer (Thermo Scientific, Germany) at the Center for Archaeology, Materials, and Applied Spectroscopy at Idaho State University, Pocatello, Idaho. Soils were dried at 55°C for 24–48 h prior to grinding soil, and BSC and soil samples in a ball mill grinder until a fine powder was attained. Samples were then packed in 5 × 9 mm silver capsules, and carbonates were removed *via* acid fumigation and then re-packed in tin. Values are reported is parts per thousand (%) relative to atmospheric N_2_ for δ^15^N using the equation: δ(%) = [(R_sample_/R_std_) − 1] × 1,000 where R_sample_ = −ratio heavy to light isotope (^15^N/^14^N or ^13^C/^12^C) of a sample. Standards of ISU Peptone, Costech Acetanilide, and DORM-3 are calibrated against international standards (IAEA-N-1, IAEA-N-2, USGS-25, USGS-40, USGS-24, and IAEA-600), and were used to create a two-point calibration.

### BSC DNA Extraction and Illumina Sequencing

Subsamples for DNA analysis were gathered from BSC samples on the same day as field collection using a flame sterilized cork borer inserted to a depth of 1 cm into each BSC until a volume of 1.75 ml was reached. The resulting materials were stored at −20°C until DNA was extracted with the PowerSoil DNA Isolation Kit (MoBio Laboratories, Inc., Carlsbad, CA, United States) following the manufacturer protocol. Extracted DNA was examined by a NanoDrop ND-1000 Spectrophotometer and a Qubit 4 fluorometer.

The eubacterial 16S rRNA gene (V3–V4 region) was amplified from all the BSC DNA samples with the primers 341F (5'-CCTACGGGNGGCWGCAG-3') and 805R (5'-GACTACHVGGGTATCTAATCC-3'; Herlemann et al., 2011). Amplicons were examined quantitatively and qualitatively and on a Fragment Analyzer (Advanced Analytical Technologies, Inc., Ames, IA, United States) before library preparation using the Nextera XT DNA Library Preparation Kit (Illumina, San Diego, CA, United States). Paired-end sequencing (2 × 300 bp) was conducted on a MiSeq Illumina platform (Illumina, San Diego, CA, United States) with a MiSeq Reagent Kit v3 at Idaho State University Molecular Research Core Facility. A total of 13,493,764 reads were obtained, with 172,030–558,823 (421,680 on average) reads per sample.

### Bacterial Community Analysis and Predictive Functional Profiling

After quality control checks using FastQC (Andrews, 2010), raw reads were demultiplexed and denoised, parsed for quality filtering and chimera removal, and clustered into operational taxonomic units (OTUs) using the package mothur (version 1.40.5; Schloss et al., 2009; Kozich et al., 2013). Briefly, VSEARCH was called for chimera removal (Rognes et al., 2016), the OptiClust algorithm was used for clustering with a similarity cutoff of 97% (Westcott and Schloss, 2017), and the RDP naïve Bayesian classifier was used against the SILVA database (release 132) with 80% bootstrap cutoff for taxonomic assignment (Wang et al., 2007). Chloroplast reads constituted 1,384 (≈1%) of algorithmically defined OTUs, and were removed preceding further analyses. Following the steps listed above, our BSC bacterial community dataset contained 124,576 OTUs from 32 observations.

To profile putative functions of BSC bacterial communities (October, 2014), we used the phylogenetic investigation of communities by reconstruction of unobserved states (PICRUSt) pipeline developed by Langille et al. (2013) coupled with and the Greengenes database (version 13.5). This pipeline estimates the functional potential of a microbial community based on 16S rRNA gene data using an extended ancestral-state reconstruction algorithm, and reports quantifiable prediction uncertainty. It takes advantage of existing annotation of gene content and 16S copy number from reference prokaryotic genomes in the IMG (Integrated Microbial Genomes and Microbiomes) database, allowing inference of metabolism pathways and genes, and taxa containing those pathways and genes. Prediction certainty is estimated by the nearest sequenced taxon index (NSTI), and the NSTI values for the analyzed BSC bacterial communities ranged from 0.16 to 0.22 (mean NSTI = 0.19 ± 0.01 s.d.), suggesting a mid-range prediction accuracy typical for soil samples (Langille et al., 2013).

We specifically focused on N-cycle pathways and bacterial taxa known to harbor various N pathways (Jetten, 2008; Nelson et al., 2016; Kuypers et al., 2018; Albright et al., 2019). The following N-cycling pathways and genes were interrogated based on their KEGG orthology (KO): *nifD* (K02586), *nifH* (K02588), *nifK* (K02591), *anfG* (K00531), *vnfD* (K22896), *vnfK*(K22897), *vnfG* (K22898), and *vnfH* (K22899) for nitrogen fixation; *amoA* (K10944), *amoB* (K10945), *amoC* (K10946), and *hao* (K10535) for nitrification; *narG*/*narZ* (K00370), *narH*/*narY* (K00371), *narI*/*narW* (K00374), *narJ*/*narV* (K00373), *napA* (K02567), and *napB* (K02568) for dissimilatory nitrate reduction to nitrite (first step of denitrification) and *nirK* (K00368), *nirS* (K15864), *norB* (K04561), *norC* (K02305), and *nosZ* (K00376) for complete denitrification steps; *nirB* (K00362), *nirD* (K00363), *nrfA* (K03385) and *nrfH* (K15876) for DNRA; *narB* (K00367), *nasA* (K00372), *nasB* (K00360), and *nirA* (K00366) for assimilatory nitrate reduction to ammonia; *glnA* (K01915), *gltB* (K00265), and *gltD* (K00266) for ammonium assimilation; and *nrtA* (K15576), *nrtB* (K15577), *nrtC* (K15578), *nrtD* (K15579), and NRT (K02575) for nitrate/nitrite transporter. The relative abundance of an individual gene in each sample was calculated as copies of that gene per average bacterial genome after considering 16S rRNA gene copies per genome. Similar strategies of N-cycling pathway inference have been previously applied to microbial communities from tallgrass prairie (Mackelprang et al., 2018), forest (Isobe et al., 2020), and crop rhizospheres (Zhu et al., 2016), where the functional predictions were considered alongside other lines of evidence. Here, the relative abundance of a putative taxon harboring an individual pathway was calculated as reads of the taxon within the whole community after considering 16S rRNA gene copies per genome.

### Statistical Analyses

Multivariate and univariate analyses were used to examine differences in the bacterial community structure and composition, as well as predicted functional traits, with respect to climate (elevation), grazing, and shrub/intershrub conditions.

Univariate responses were evaluated within the split-split plot experimental framework using conventional mixed-effect model approaches (Aho, 2016, Ch. 10). These models used the Satterthwaite procedure for computing error degrees of freedom for fixed-effects tests and likelihood-ratio tests for random effects. The models were codified so that the largest factor, (with respect to a split plot hierarchy) in which multiple levels were measured, was defined as a random blocking variable. This was necessary because, under our sampling scheme, the levels of such a factor were unreplicated (see Figure 2). Mixed-effect models that failed to converge at default tolerances are not described here, but can be found in Supplementary Material (Supplementary Table S1).

Multivariate genetic analyses of the split-split plot experimental design required application of multiple analyses (see Supplementary Material). Whole plot (elevation) effects were examined within the context of a randomized block design within date blocks. Multiway factorial structures were designated for split plot (grazing) and split-split plot (shrub/intershrub) levels (see Supplementary Material). Multivariate tests of the null hypothesis of no BSC community differences were conducted using the PERMANOVA approach of Anderson (2001).

We graphically depicted multivariate relationships of samples in BSC community space using nonmetric multi-dimensional scaling (NMDS; Kruskal, 1964). Bray-Curtis dissimilarity (Bray and Curtis, 1957) was used for multivariate procedures requiring resemblance matrices, e.g., PERMANOVA and NMDS. Correlations of important biotic and abiotic variables to NMDS projections were determined using the vector fitting method of Oksanen et al. (2019).

Indicator species analysis (ISA; Dufrene and Legendre, 1997) was used to test for the association of taxa designations to elevation, grazing, and shrub levels. Taxa were required to occur at two or more samples for inclusion in ISA analyses (e.g., Aho et al., 2020). Unclassified taxa were eliminated from analyses. Values of *p* for tests of the null hypothesis that associations between taxa and categorical explanatory levels were no better than random were based on 1,000 permutations.

We used the R statistical environment (version 3.2.0; R core team [https://www.R-project.org]) for all statistical analyses, with heavy reliance on the packages vegan (Oksanen et al., 2019) and asbio (Aho, 2019) for multivariate and graphical applications, and the packages lme4 (Bates et al., 2015) and lmerTest (Kuznetsova et al., 2017) for the mixed-effect models. In addition, the package phyloseq was used for processing sequencing reads. A significance level of *α* = 0.05 was used for statistical tests.

### Availability of Sequence Data

All Illumina reads are deposited at the NCBI Sequence Read Archive (SRA), under the BioProject accession number PRJNA633217 (https://www.ncbi.nlm.nih.gov/bioproject/PRJNA633217).

## Results

### Soil and BSC Characteristics

**Figure 3:**
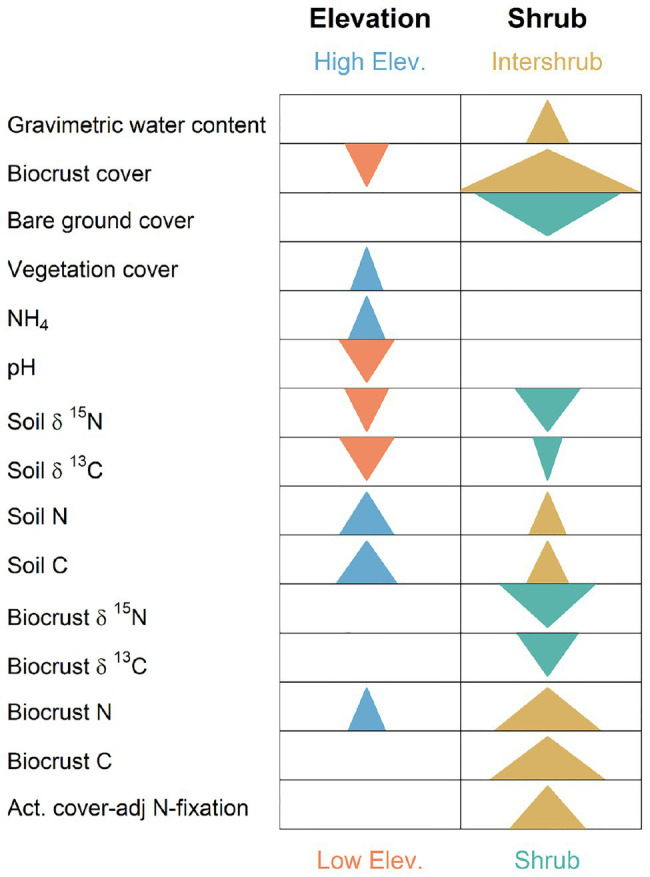
Graphical summary of significant split-split-plot analyses (or in the absence of date replication), split-plot analyses for biocrust, and soil biogeochemistry response variables. Arrows indicate that statistically significant main effects occurred for elevation or shrub presence/absence in a mixed model along with an absence of significant interactions with other factors (see Supplementary Table S1). Arrows point in direction of significant numeric increase. Arrow width is scaled by the log of the inverse *p*. Thus, wider arrows denote smaller values of *p*, and indicate more dramatic changes.

Soil and biocrust characteristics varied with the predictors under consideration, particularly elevation and the presence/absence of shrub canopy. Directional trends for characteristics that varied significantly with respect to main effects (elevation, grazing, and shrub presence/absence) and lacked significant interactions are summarized in Figure 3. Note that no response variable was significantly associated with grazing in the absence of significant interactions of grazing with other factors (Figure 3). Nuanced interactions between grazing, shrub presence/absence, and elevation with respect to BSC nitrogenase activity at the study sites have been described in Schwabedissen et al. (2017). Supplementary Material provides summaries for all mixed effect models (Supplementary Table S1), and provides centrality and dispersion estimates for all biochemical variables, across all predictor levels (Supplementary Tables S2−S6).

Soil and biocrust abiotic/biogeochemistry variables whose values increased most strongly with elevation included: soil N ($X_{1}^{2}=$ 3.28, *p* = 0.005, Figure 3) with mean values ranging from 0.07% at the lowest elevation (F) to 0.44% at the highest elevation site (MBS); soil C ($X_{1}^{2}=$ 3.28, *p* = 0.005), which increased from 0.67 to 5.7% from F to MBS; and NH_4_ (*F*_1,291_ = 3.19, *p* = 0.024), which ranged from 0.9 to 3.8 μg g^−1^ dry soil. Strongest decreasers with elevation included pH (*F*_3,6.2_ = 12.48, *p* = 0.005, Figure 3), which ranged from 7.6 to 6.0 from F to MBS; biocrust cover ($X_{1}^{2}=$ 6.2, *p* = 0.013), which decreased from 58.1 to 1%; soil δ^15^N ($X_{1}^{2}=$ 6.14, *p* = 0.013), which decreased from 4.7 to 0.13% (AIR); and soil δ^13^C ($X_{1}^{2}=$ 7.78, *p* = 0.005), which decreased from −21.6 to −26.8% (PDB; also see Supplementary Table S1).

Strongest biochemical/abiotic increasers in intershrub spaces were biocrust cover (*F*_1,70_ = 35.2, *p* = 1.0 × 10^−7^) with intershrub and shrub means of 50.2 and 26.2%, respectively; biocrust N (*F*_1,70_ = 17.96, *p* = 6.8 × 10^−5^) with intershrub and shrub means of 0.49 and 0.29%, respectively; biocrust C (*F*_1,70_ = 19.59, *p* = 3.4 × 10^−5^) with intershrub and shrub means of 7.7 and 3.7%, respectively; and actual N fixation (*F*_1,70.8_ = 11.5, *p* = 1.5 × 10^−5^) with intershrub and shrub means of 66.6 and 55.3 kg ha^−1^ year^−1^, respectively. Variables strongly decreasing in intershrub spaces included bare ground cover (*F*_1,70_ = 26.5, *p* = 2.3 × 10^−6^) with shrub and intershrub means of 50.1 and 25.1%, respectively; biocrust δ^15^N (*F*_1,70_ = 15.7, *p =* 0.0002) with shrub and intershrub means of 5.1 and 3.7% (AIR), respectively; and soil δ^15^N (*F*_1,70_ = 10.1, *p* = 0.002) with shrub and intershrub means of 7.3 and 6.8% (AIR), respectively (also see Supplementary Table S1).

### BSC Bacterial Communities in Cold Sagebrush Steppe Ecosystems

#### OTU Richness and α-Diversity

**Figure 4:**
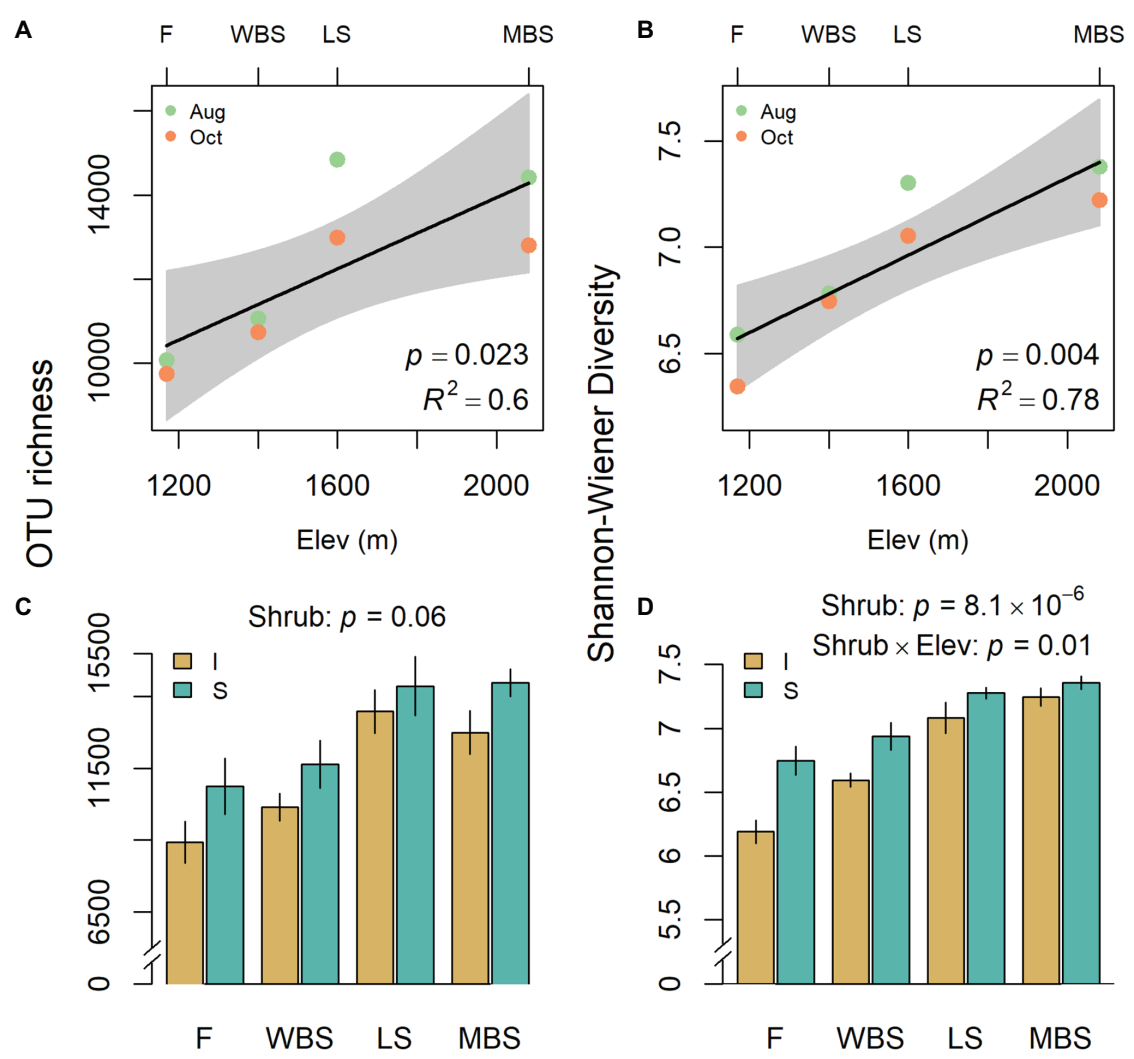
Variation in operational taxonomic unit (OTU) richness **(A,C)** and Shannon diversity **(B,D)** with respect to elevation **(A,B)** and shrub/intershrub levels **(C,D)**. Shading around regression fits in **(A,B)** are 95% confidence intervals for true fitted values. Errors in **(C,D)** are SEMs.

Biological soil crust bacterial communities shifted with shrub/intershrub levels, and particularly along the elevation gradient, regardless of sampling date (Figure 4). Shannon diversity and richness both increased linearly with elevation, when elevation was treated as a quantitative variable (Figures 4A,B) and within our split-split plot framework where elevation was treated as a categorical variable: *F*_3,15_ = 6.9, *p* = 0.004 and *F*_3,3_ = 58.8, *p* = 0.004 for richness and diversity, respectively. OTU richness did not vary significantly with shrub/intershrub levels (*F*_1,15_ = 4.0, *p* = 0.064; Figure 4C). However, Shannon diversity was higher under shrub canopies than in shrub interspaces (*F*_1,12_ = 55.03, *p* = 8.1 × 10^−6^; Figure 4D). A significant interaction occurred between elevation and shrub/intershrub levels in the Shannon diversity mixed-effects model (*F*_3,12_ = 5.74, *p* = 0.01), such that shrub/intershrub diversity levels became more similar with increasing elevation (Figure 4D). Nevertheless, elevation and shrub/intershrub main effects remained interpretable, because OTU diversity under shrub canopies remained higher than intershrub OTU diversity for all the elevations (Figure 4D; Aho, 2016, p. 461–462). Grazed and ungrazed sites did not have significantly different levels of OTU richness (*F*_1,15_ = 0.04, *p* = 0.85) although a non-significant trend (grazed > ungrazed) was evident for Shannon diversity (*F*_1,12_ = 4.68, *p* = 0.051).

#### OTU β-Diversity

A split-split-plot PERMANOVA identified elevation (*F*_3,3_ = 7.7, *p* = 0.001) and shrub/intershrub levels (*F*_1,8_ = 3.7, *p* = 0.01) as important determinants of BSC bacterial community composition (Table 2). No other main factors considered in this study were significantly associated with patterns of OTU composition (Table 2). A general lack of significant grazing effects prompted us to focus on elevation and shrub canopy effects on BSC communities in subsequent multivariate analyses.

**Table 2:** Effects of main factors on BSC bacterial community composition under a split-split-plot design.

	Df	SS	MS	*F*	*p*
**Whole-plot**					
Date (block)	1	0.055	0.055	2.273	0.159
Elevation	3	0.558	0.186	7.666	**0.001**
Whole-plot error	3	0.073	0.024		
**Split-plot**					
Grazing	1	0.049	0.049	1.090	0.345
Grazing × Elevation	3	0.177	0.059	1.317	0.274
Split-plot error	4	0.179	0.045		
**Split-split-plot**					
Shrub	1	0.323	0.323	3.666	**0.010**
Shrub × Grazing	1	0.053	0.053	0.600	0.832
Shrub × Elevation	3	0.277	0.092	1.047	0.405
Shrub × Grazing × Elevation	3	0.189	0.063	0.713	0.820
Split-split-plot error	8	0.706	0.088		
Total	31				

Statistically significant PERMANOVA results are bolded.

**Figure 5:**
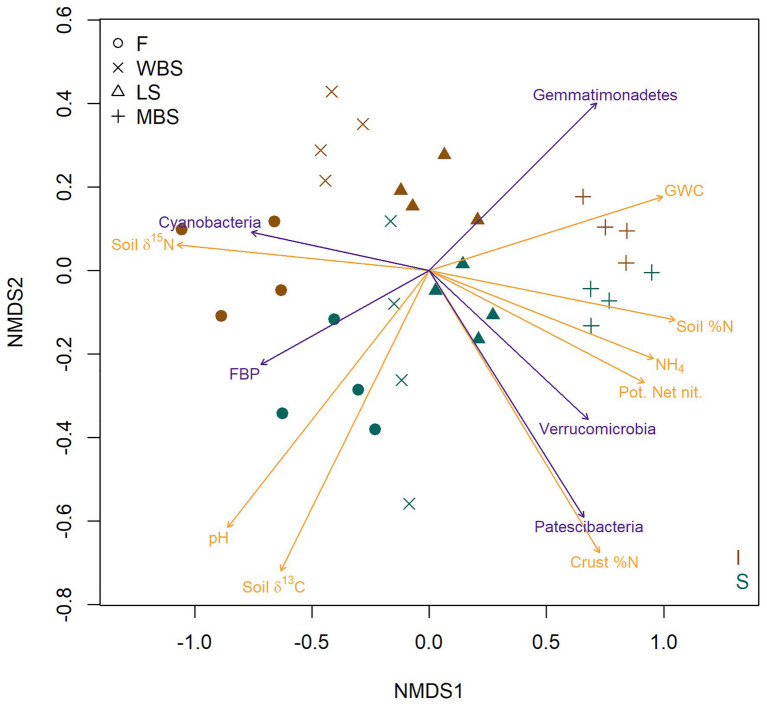
Nonmetric multi-dimensional scaling (NMDS) ordination (stress = 0.089) based on Bray-Curtis dissimilarities of BSC community OTU composition. Red symbols represent BSCs from understory shrubs (S); black symbols represent BSCs from intershrub spaces (I). Arrows delineate vector fits of strongly significant (*α* = 0.001) biogeochemical variables (brown arrows) and bacterial phyla (green arrows) that are correlates of the NMDS projection. Arrow direction indicates the direction of most rapid increase for a denoted variable in the projection. Arrow length is scaled by the strength of association (*R*^2^) of a multiple regression in which ordination axes serve as predictors for the variable indicated with an arrow. GWC, gravimetric water content; Pot. Net. Nit., potential net nitrification.

The strong effect of elevation and shrub canopies on BSC bacterial communities is strikingly illustrated in a two-dimensional NMDS ordination of OTU composition (Figure 5). Dimension one (NMDS 1) clearly separates samples along an elevational gradient from the lowest (F) to the highest (MBS), whereas dimension two (NMDS2) separates BSC bacterial communities in shrub understories from those in intershrub spaces (Figure 5). When overlaid on the ordination, vector fitting results showed strongest (permutation *p* < 0.01) non-redundant correlations to the point projection with respect to measured soil biogeochemical variables (brown arrows) and several bacterial phyla (green arrows). As indicated in other analyses above, lower altitude (F) soils were alkaline and enriched in ^15^N and ^13^C, with δ^13^C being particularly enriched under shrub canopies (Figure 5). Lower elevation BSCs, particularly under shrubs, were also enriched in δ^13^C. High altitude (MBS) soils were Mollisols with deep, high organic matter and nutrient enriched surface soils, showing higher potential net nitrification and relatively high levels of soil N and NH_4_^+^. Higher elevation biological crusts under shrubs had the highest levels of soil N (Figure 5). Vector fitting further indicated that lower altitude biocrusts were dominated by the phylum Cyanobacteria in intershrub spaces and FBP under shrubs, whereas higher altitude BSCs were indicated dominated by Patescibacteria and Verrucomicrobia under shrubs, and by Gemmatimonadetes in intershrub spaces (Figure 5).

#### Taxonomic Composition

A total of 26 bacterial phyla, 63 classes, 155 orders, 266 families, and 464 genera were observed in all the BSC samples (Supplementary Materials; Supplementary Table S9). The five most abundant BSC phyla at the study sites were Cyanobacteria (11.0%), followed by Actinobacteria (8.5%), WS2 (7.0%), Acidobacteria (6.3%), and Nitrospirae (6.2%; Supplementary Table S10). Unknown/unclassified/uncultured taxa made up 1.6, 9.3, 11.0, 13.3, and 17.2% of total reads at the phylum, class, order, family, and genus level, respectively (Supplementary Tables S10−S14).

### Indicator Bacterial Taxa

Indicator species analysis results showed 58% of phyla, 54% of classes, 45% of orders, 44% of families, and 41% of genera were statistically significant indicators of one of the four elevation zones (Supplementary Table S16). After controlling false discovery rate (FDR; Benjamini and Hochberg, 1995), these percentages fell to 46, 39, 29, 32, and 28%, respectively (Supplementary Table S16).

**Figure 6:**
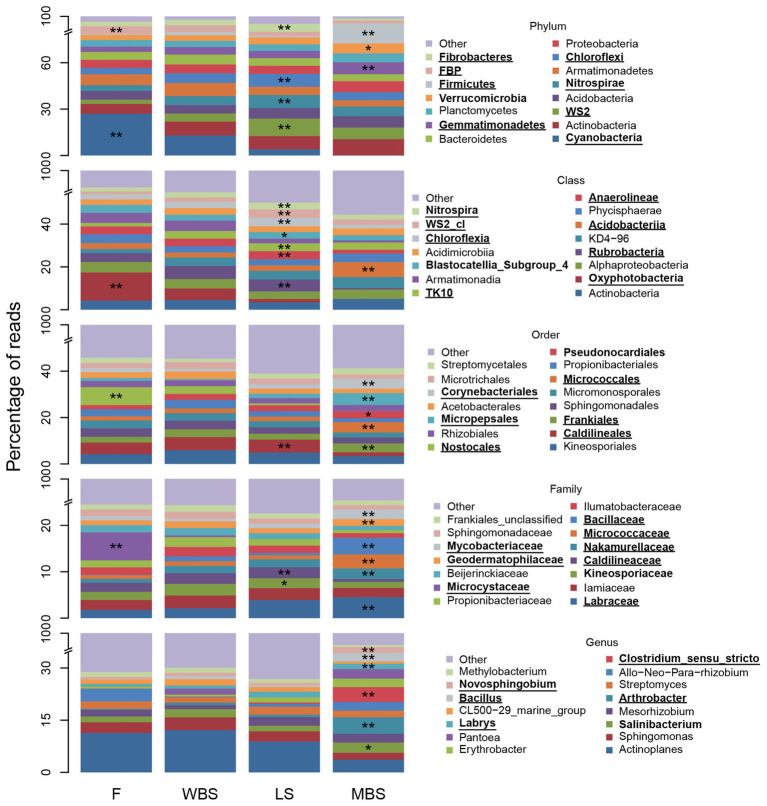
Fifteen most abundant BSC bacterial taxa and indicators, at positions along the elevational gradient. The “other” category comprises all other less abundant taxa and “unclassified” and “uncultured” groups. Asterisks (*) denote a significant indicator species analysis (ISA) taxon and are accompanied by bold font entries in the legend. Double asterisks (**) denote a significant ISA taxon after controlling for false discovery rate (FDR) and are accompanied by bold text and underlined entries in the legend.

Strong variations in BSC indicator taxa with elevation were consistent with and reflected the major shifts in OTU composition shown along the first dimension of the NMDS ordination in Figure 5. Figure 6 depicts relative abundance of the 15 most abundant BSC taxa at varying levels across elevational zones, and displays ISA results for those taxa. Significant BSC indicators (after FDR control) of the lowest elevational zone (F) included Cyanobacteria and FBP (phylum), and Cyanobacterial subdivisions including Oxyphotobacteria (class), Nostocales (order), and Microcystaceae (family). The WBS elevational zone had few significant BSC indicators, and none for taxonomic hierarchies more general than order (Supplementary Table S17). LS BSCs were significantly indicated by Chloroflexi, Fibrobacteres, Nitrospirae, and WS2 (phylum), Anaerolineae, Chloroflexia, Nitrospira, TK10, an unclassified WS2 class, Rubrobacteria (class), Caldilineales (order), and Caldilineaceae (family). The highest elevation zone, MBS, had the highest number of significant indicators (Supplementary Table S17), signifying that BSC communities there were highly diverse and distinct from those of other elevations. Significant MBS BSC indicators included Firmicutes and Gemmatimonadetes (phylum), Acidobacteriia (class), Corynebacteriales, Frankiales, Micrococcales, and Micropepsales (order), Bacillaceae, Geodermatophilaceae, Labraceae, Micrococcaceae, Mycobacteriaceae, and Nakamurellaceae (family). Interestingly, the 15 most abundant genera were not significant indicators at lower elevations. In contrast, many of these abundant genera were significant indicators at the highest elevation, MBS, including *Arthrobacter*, *Bacillus*, *Clostridium* (*sensu stricto*), *Erythrobacter*, *Labrys*, and *Novosphingobium* (genus). Additional indicator taxa with less relative abundance were also observed (complete ISA results are shown in Supplementary Tables S18–S20).

Indicator species analysis results showed 42% of phyla, 38% of classes, 38% of orders, 32% of families, and 24% of genera were statistically significant indicators of shrub or intershrub BSCs (Supplementary Table S22). After controlling FDR, these percentages fell to 25, 20, 19, 16, and 10%, respectively (Supplementary Table S22). Numbers of significant indicators were markedly higher in shrub understories than in intershrub spaces (Supplementary Table S23).

**Figure 7:**
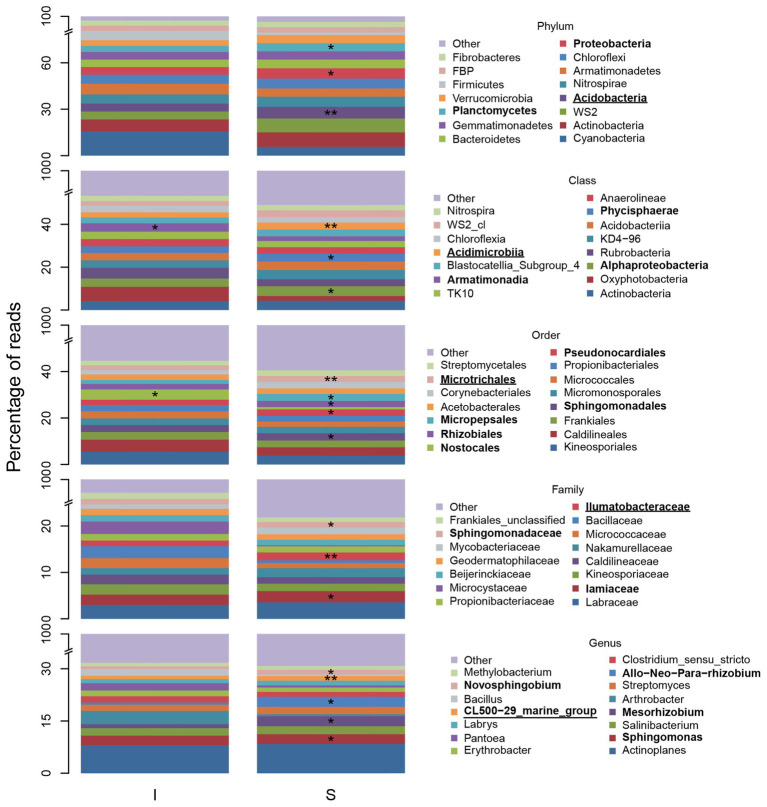
Fifteen most abundant BSC bacterial taxa and indicators, at varying levels, understory shrubs (S) or in intershrub spaces (I). The “other” category comprises all other less abundant taxa and “unclassified” and “uncultured” groups. *Allorhizobium*-*Neorhizobium*-*Pararhizobium*-*Rhizobium* was abbreviated. Asterisks (*) denote a significant ISA taxon and are accompanied by bold font entries in the legend. Double asterisks (**) denote a significant ISA taxon after controlling for FDR and are accompanied by bold text and underlined entries in the legend.

Figure 7 shows relative abundance of the 15 most abundant BSC taxa at shrub/intershrub zones, along with ISA results for those taxa. Complete ISA results for shrub levels are provided in Supplementary Material (Supplementary Tables S24–S26). Among the 15 most abundant taxa, none was a significant indicator for intershrub spaces after FDR control. Among all the less abundant taxa, statistically significant indicators for intershrub spaces were chiefly Deinococcus-Thermus (phylum) and its subsets Denoococci (class), Deinococcales (order), Deinococcaceae (family), and *Deinococcus* (genus), as well as the genus *Sporosarcina*. Many more indicator taxa were observed under shrub canopies than in shrub interspaces (Supplementary Table S23). Among the 15 most abundant taxa, significant shrub indicators included Acidobacteria (phylum), Acidimicrobiia (class), Microtrichales (order), Ilumatobacteraceae (family), and the *CL500-29* genus under Actinobacteria (genus; Figure 7). Among other less abundant taxa, there were even more indicators of shrub BSCs (Supplementary Tables S24–S26).

### Bacterial Taxa Related to Nitrogen Cycling

**Figure 8:**
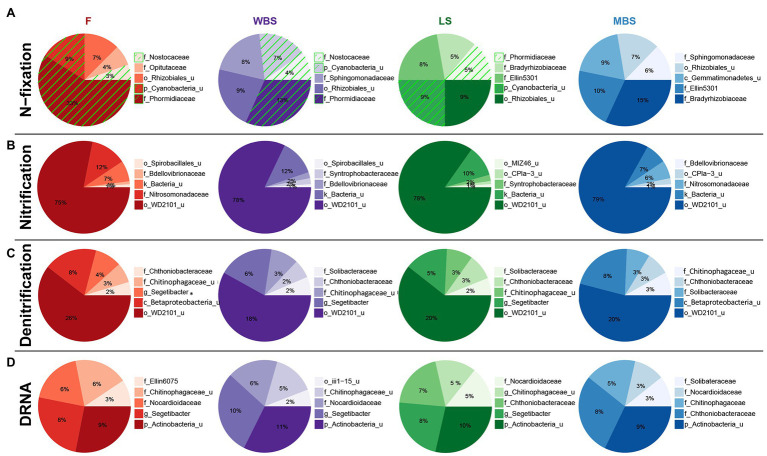
Top five family-level functional designees across elevation levels for **(A)** N-fixation (cyanobacterial taxa highlighted by green hatching), **(B)** nitrification, **(C)** denitrification, and **(D)** dissimilatory nitrate reduction to ammonia (DNRA). Area of each pie slice is proportional to the family’s percentage among the top five families. Numbers in the text are percentages of the families with respect to all taxa in the communities. “_u” = “unclassified”; “g_Segetibacter” = “f_Chitinophagaceae_g_Segetibacter.”

Functional inference from PICRUSt, when considered alongside other analyses, suggested the presence of distinct N-fixing communities along the elevational gradient. Cyanobacterial taxa dominated the lower elevations (F, WBS) and were absent at the highest elevation (MBS; Figure 8A). Rhizobial taxa replaced Cyanobacteria at higher elevations (LS and MBS), particularly the family Bradyrhizobiaceae and an unclassified family of the Rhizobiales order. In parallel with this fundamental shift in the N-fixing communities, PICRUSt estimated an overall decrease in the relative abundance of nitrogenase genes (predominantly *nifD*, *nifK*, and *nifH*) along the elevation gradient (Supplementary Figure S5). This observation was supported by ISA results, which showed that alongside general community shifts, dramatic shifts in BSC diazotrophic taxa occurred with elevation and shrub/interspace patches. These included: (1) the replacement of Cyanobacteria with putative N-fixing bacteria that frequently form symbiosis with plant species, i.e., Rhiyzobiales and Frankiaceae, with increasing elevation (Supplementary Table S19) and (2) Cyanobacterial taxa, e.g., Nostocales, being replaced by rhizobia N-fixers, e.g., *Allorhizobium*-*Neorhizobium*-*Pararhizobium*-*Rhizobium* and *Mesorhizobium* under shrub canopies (Figure 7). This finding was further supported by analysis of the *nifH* gene diversity using terminal restriction fragment length polymorphism (TRFLP), which suggested that *nifH* genes were present in a subset of the aforementioned bacterial taxa (data not shown).

A variety of bacterial taxa potentially involved in nitrification (Figure 8B), denitrification (Figure 8C), and dissimilatory nitrate reduction to ammonium (DNRA; Figure 8D) were observed, and their distribution was similar across elevations. Some of these taxa have known roles in N cycling, such as ammonia oxidizers in the Nitrosomonadaceae family (Arp et al., 2007), while some of these taxa were previously identified in water treatment facilities with N removal function, such as the family Chthoniobacteraceae and Chitinophagaceae (Shu et al., 2015; Gao et al., 2019).

**Figure 9:**
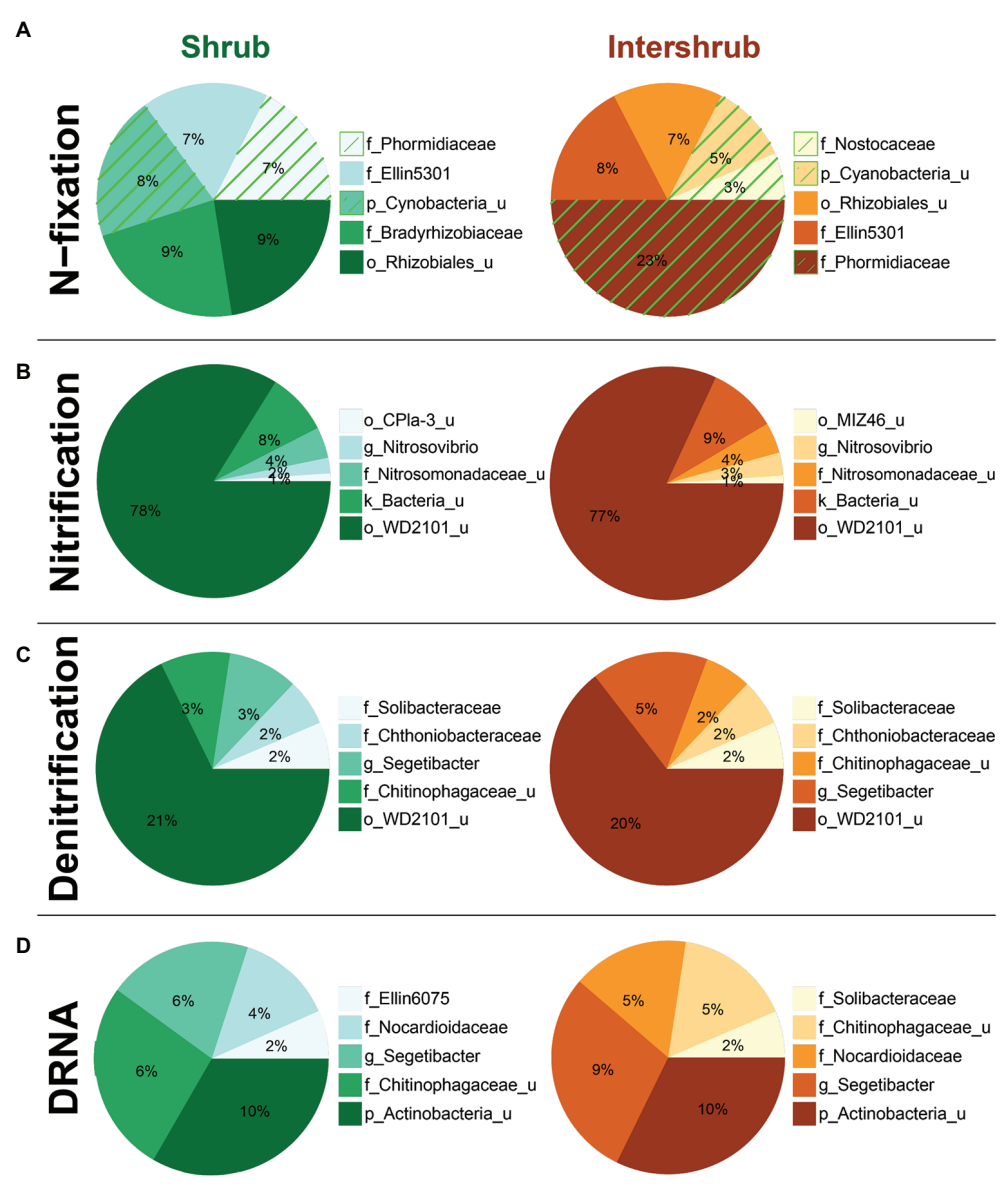
Top five family-level functional designees across shrub/intershrub levels for **(A)** N-fixation (cyanobacterial taxa highlighted by green hatching), **(B)** nitrification, **(C)** denitrification, and **(D)** dissimilatory nitrate reduction to ammonia (DNRA). Area of each pie slice is proportional to the family’s percentage among the top five families. Numbers in the text are percentages of the families with respect to all taxa in the communities. “_u” = “unclassified”; “g_Segetibacter” = “f_Chitinophagaceae_g_Segetibacter”; “g_Nitrosovibrio” = “f_Nitrosomonadaceae_g_Nitrosovibrio.”

With respect to shrub/intershrub levels, putative N-fixing taxa included Cyanobacteria taxa (e.g., mostly the family Phormidiaceae and to a less extent also the family Nostocaceae) drove BSC N-fixation at intershrub zones, particularly at lower elevations, whereas putative N-fixing taxa under shrub canopies included rhizobial taxa (e.g., the family Bradyrhizobiaceae; Figure 9A). In contrast, similar bacteria potentially involved in nitrification (Figure 9B), denitrification (Figure 9C), and DNRA (Figure 9D) were seen at shrub/intershrub zones.

## Discussion

### Elevation/Climate and BSC Communities

Our study area spanned a wide range of climatic conditions typical of the intermountain region of the western United States, driven by nearly 1,000 m change in elevation. Along this gradient, and in accordance with hypothesis 1a, putative N_2_-fixing bacterial taxa, particularly those belonging to the phylum Cyanobacteria, varied with elevation/climate. Specifically, cyanobacteria – which were dominant in drier, warmer, and alkaline soils at lower elevations with abundant biocrusts – were replaced by putative symbiotic N_2_ fixers at higher elevations and in wetter and cooler climates (Figures 3, 5, 6, 8). Our findings accord with Hagemann et al. (2015), who found that abundances of cyanobacteria changed along a precipitation gradient. Increases in the abundance of putative symbiotic N-fixers followed increases in total vegetation cover and decreases in BSC cover with elevation (Figure 3). Thus, increases in putative symbiotic diazotrophs are likely due to the association of these taxa with grass and shrub roots.

Despite decreasing BSC cover, BSC microbial diversity surprisingly increased with elevation (Figure 4), which coincided with the decrease in soil pH and increase in soil ammonium (Figure 5). These findings are contradictory to Blay et al. (2017) who found decreasing diversity at the phylum level along this elevation gradient, but not at the genus level. Differences in findings associated with this study and Blay et al. (2017) can be explained by several improvements made here, including different amalgamation methods (volume in this study vs. DNA mass-based), repeat sampling, better library preparation (e.g., more optimal conditions), and deeper sequencing and higher taxonomic resolution. Indeed, unclassified phyla dropped from 26% in Blay et al. (2017) to 1.6% in this study. Additionally, OTU-based community diversity as reported here better reflects true diversity. Studies along elevation gradients in other regions have shown mixed effects on microbial diversity. Consistent with our study, Yang et al. (2014) showed increases in gene diversity with elevation, and associated this with aboveground productivity and soil edaphic characteristics, especially lower pH and higher nutrient availability as ammonium. In contrast, Shen et al. (2013) showed variable response in bacterial diversity with elevation, but again showed strong controls of pH on diversity. More recently, Li et al. (2018) reported a stair-step pattern of soil bacterial diversity along elevation in a mountain ecosystem, including an abrupt decrease between 2,600 and 2,800 m, but no significant change at either lower (1800–2,600 m) or higher (2800–4,100 m) elevations. The same report showed significant shifts in bacterial community structure from the lower to the higher elevations and attributed the changes to shifts in soil pH and vegetation types that were also affected by climate change. They further suggested that environmental filtering played an overwhelming role in the assembly of bacterial communities, with contributions from spatial attributes, likely due to environmental heterogeneity, increased at higher elevations.

In this study, moss-dominated BSCs were more common than lichen-dominated BSCs at all elevations. This is partly explained by mass; however, mosses grow and colonize areas more quickly than lichens (Dettweiler-Robinson et al., 2013). Interactions between mosses and lichens can result in moss positively affecting lichen cover during early-succession of BSCs. These interactions, however, become negative as competition increases once BSC cover becomes higher with late successional BSCs (Dettweiler-Robinson et al., 2013). This interaction, along with the increase in shrub and grass cover (Ochoa-Hueso et al., 2011; Elliott et al., 2014), may explain why most of the BSCs at our sites were associated with moss within the shrub canopy.

The shift from cyanobacterial to symbiotic N_2_-fixing genera that potentially mediate N_2_ fixation along the climatic gradient may suggest that the structure of BSC N_2_-fixing communities could change under a warming climate. Warming of 1.4–2.3°C in mean annual minimum temperature and 0.8–1.4°C in mean annual maximum has been documented over a 40 year (1965–2006) period for the study area (Nayak et al., 2010; Seyfried et al., 2011). Precipitation during this time period showed no significant changes but the phase of precipitation changed. Specifically, water year precipitation as snow decreased by 4–5% per decade at mid elevation sites, down from 53 to 38%, and decreased from 41 to 22% at lower elevation sites. Temperatures are expected to increase 1.5–4°C in the western United States and changes in precipitation phase are projected to continue such that parts of the Intermountain West will become snow free (Klos et al., 2014) with accompanying shifts in timing of sagebrush productivity (Flerchinger et al., 2019). Muñoz-Martín et al. (2019) documented shifts in cyanobacterial composition along environmental gradients in Spain and pointed to a potential replacement of cool-adapted by warm-adapted nitrogen-fixing cyanobacteria (such as *Scytonema*) and switches in dominance by *Microcoleus vaginatus* to thermotolerant, bundle-forming cyanobacteria. The effects of climatic changes on BSCs in southeastern Utah, which is currently dominated by rain in late summer and early autumn with infrequent winter rain or snow (Johnson et al., 2012), has been previously examined (Ferrenberg et al., 2015; Steven et al., 2015). Ferrenberg et al. (2015) showed an increase in cyanobacteria and a decrease in moss and lichen cover with warming, an increase of small summer rainfall events, and the combination of both. However, when increased temperatures and shifts in summer precipitation were combined, Steven et al. (2015) found that cyanobacteria abundance decreased. Within our study sites, precipitation is higher than in southeastern Utah, and occurs more often as snow at the highest (coldest) elevation. At our highest elevations, cyanobacteria are essentially absent in BSCs. Conversely, precipitation at our lower (warmest) elevation sites is less frequent and occurs more often as rain, and the BSCs are dominated by cyanobacteria. Given regional precipitation and temperature projections (Klos et al., 2014), we might expect a decline in lichens, mosses, and cyanobacteria at the warmest elevations, whereas in the snow-dominated, cooler elevations, we would anticipate increased cyanobacterial abundance. Cyanobacterial taxa have been suggested to be N-cycling specialists, primarily carrying the N fixation and assimilatory nitrate/nitrite to ammonium pathways (Nelson et al., 2016), as well as the incomplete denitrification pathway (Albright et al., 2019). Changes in their abundance could lead to N-cycling imbalance in BSCs.

Importantly, ISA results suggested an increased reliance on nodulated, symbiotic N-fixation at the study sites with increasing elevation. These included the increased presence of Rhizobiales *incertae sedis* (Supplementary Table S19), a bacterial family whose species are known to form an endosymbiotic N-fixing association with roots of plants in Fabaceae (legumes), and Frankiales, a bacterial order whose taxa are known to forms symbiosis with actinorhizal plants, including species in the families Betulaceae (birches) and Rosaceae (Trivedi et al., 2020). The increased presence of these groups with elevation can reflect increasing plant cover with elevation, including legumes like *Lupinus* sp. (lupine) which visually dominate open slopes at the study sites during much of the growing season (M. Seyfried USDA, personal communication). Additionally, NMDS coupled with vector fitting identified several phyla and biogeochemical variables as strong explanatory factors for the observed variations in biocrust bacterial communities. Verrucomicrobia that are known as nutrient-scavenging and can encodes a wide range of carbohydrate degradation enzymes (Martinez-Garcia et al., 2012) was more represented in shrub BSCs at higher elevations where soils were nutrient enriched (Figure 5). Interestingly, the newly defined super phylum Patescibacteria, known to thrive under oligotrophic conditions (Tian et al., 2020), is also correlated with shrub BSCs at higher elevations. Consistent with increasing soil NH_4_^+^ along the elevational gradient, ureolytic bacteria such as Micrococcales and Corynebacteriale (Wang et al., 2018) were identified as significant indicators at the highest elevation (Figure 6). Moreover, Gemmatimonadetes was increasingly abundant in BSCs at higher elevations (Figure 5). However, it correlated more with intershrub spaces than with shrub canopies, in contrast to a previous report of this phylum as an indicator of rhizospheres in semi-arid ecosystems (Rodríguez-Caballero et al., 2017). The candidate phylum FBP, often found in phyllosphere samples (Ruiz-Pérez et al., 2016), was more representative of lower elevation BSCs, but its ecological functions are poorly understood (Tahon et al., 2018). While our data clearly show correlations between bacterial taxa and environmental variables, the exact ecophysiology and function of these taxa remains obscure. Future research using multiple omics approaches, particularly metatranscriptomics and metaproteomics, may provide new insights.

### Grazing and BSC Communities

Counter to hypothesis 1b, grazing did not affect BSC community diversity or composition (Table 2; Figures 4, 5). Unlike studies of pinnacled and rugose biocrusts in other regions with distinct soil and vegetation conditions (Belnap, 2002; Yeager et al., 2004; Dojani et al., 2013), the genus *Microcoleus* was rare in rolling biocrust communities in our study area. The genus *Nostoc*, which often occurs in more mature biocrusts (Barger et al., 2016), was more abundant at several ungrazed sites, but there was no significant, consistent trend across elevations in both shrub and intershrub locations. Overall, while chronic physical disturbance has been found to alter pinnacle and rugose biocrust communities in arid shrublands and biocrust communities in grass-shrub steppe, with Cyanobacteria species being negatively impacted (Belnap, 2003; Yeager et al., 2004; Dojani et al., 2013; Velasco Ayuso et al., 2020), our data showed insignificant effect of grazing on rolling biocrusts in cold sagebrush steppe ecosystems. Notably, another study of biocrusts in warm semi-arid to dry-subhumid woodland found that grazing promoted biocrust functional diversity through an increase in biocrust richness concomitant with increasing vascular plant richness (Mallen-Cooper et al., 2018). Therefore, we posit that grazing effects on biocrust diversity may be indirect, or dependent on aridity and vegetation.

### Shrub Patches and BSC Communities

In accordance with hypothesis 1c, we found higher abundance of cyanobacteria at intershrub locations compared to understory shrub-canopies (Figure 5). As elevation increased, cyanobacteria decreased dramatically within intershrub spaces. Similar BSC changes have been documented in the Kalahari Desert, where large reductions in cyanobacteria were attributed to strong niche partitioning of the microbial community between different vegetation types (Elliott et al., 2014). We posit that decreasing cyanobacterial abundance in intershrub areas at our study sites was due to vegetation cover increases with higher precipitation at the upper elevations. This spatial heterogeneity in diversity, abundance, and potential function of BSC systems is an important consideration in the management of invasive grasses that encroach on intershrub spaces, as *Bromus tectorum* negatively impacts BSC cover (Dettweiler-Robinson et al., 2013).

Despite higher abundance in intershrub spaces, Cyanobacterial shifts with shrub/intershrub levels were not readily apparent in ISA results (Figure 7). This is likely due to the fact that these analyses excluded cyanobacterial taxa that were unclassified at Class or lower levels. Nevertheless, ISA results suggested a preference of taxa that were potentially nodulating N-fixers for shrub understories (Figure 7; Supplementary Table S25). Statistically significant shrub BSC indicators included the order Rhizobiales, and the genus designation *Allorhizobium*-*Neorhizobium*-*Pararhizobium*-*Rhizobium* that are known to be associated with plant roots (Supplementary Tables S25, S26; Mangeot-Peter et al., 2020; Trivedi et al., 2020). The higher presence of rhizobia in BSCs under canopies could be associated with a number of factors including higher levels of plant root exudates which may attract rhizobia (Currier and Strobel, 1976), and spatial associations of rhizobia to highly productive areas due to continual breakdown of legume organic matter. Projected changes in precipitation phase and accompanying shifts in timing of sagebrush productivity in this region (Klos et al., 2014; Flerchinger et al., 2019) may lead to changes in shrub-associated N-fixing communities and resulting N cycling processes.

### Taxa Potentially Involved in Nitrogen Cycling Along a Climatic Gradient

As a first step to understand potential ecological functions of the biocrust microbial communities in the study ecosystem, we used the PICRUSt pipeline to infer putative N-cycling functional trends. Similar taxa that putatively mediate nitrification, denitrification, and DNRA groups were observed across elevations, as well as in shrub and intershrub areas. Nitrogen-fixing groups, however, were distinct with respect to elevation and shrub canopy association, which supports hypothesis 2. There was a clear shift from cyanobacterial taxa at lower elevations to rhizobial taxa at higher elevations and to rhizobial taxa at the highest elevation. These observations align with diversity and ISA results, and reinforce climate and vegetation as important controls on the assembly of biocrust bacterial communities in this cold sagebrush steppe ecosystem.

Studies have shown that N fixation increases as biocrust develops, and that cyanolichen biocrusts (*Collema* spp., *Nostoc commune*) have the highest measured N-fixation activity, followed by moss biocrusts and dark cyanobacterial biocrusts (dominated by a mix of *Nostoc* spp. Tolypothrix spp., *Scytonema* spp. and *Microcoleus* spp.). Light cyanobacterial biocrusts (dominated by *Microcoleus* spp.) have the lowest measured N-fixation activity associated with heterotrophic diazotrophic bacteria (Pepe-Ranney et al., 2016; Barger et al., 2016; Bowker et al., 2018). While moss-dominated BSCs were common at all the study sites, the variations in putative N-fixing taxa along the elevational gradient likely affect N cycling processes. Indeed, Schwabedissen et al. (2017) previously found higher nitrogenase activity of BSCs in warmer, drier climates at lower elevations than in colder, wetter climates at higher elevations in this ecosystem. These trends in nitrogenase activity may be attributed to a shift from cyanobacterial to rhizobial N fixers and a decrease in the relative abundance of nitrogenase genes (predominantly *nifHDK*) with elevation, as predicted by functional inference here. It should be emphasized that the accuracy of 16S based functional inference relies on the availability of reference genomes and accurate genome annotation. Currently, sequenced genomes of environmental isolates are still limited, and cryptic N-cycling pathways may exist, both affecting the use of marker-gene approaches to quantify N-cycling potential (Albright et al., 2019). Moreover, compared to N fixation and nitrification pathways, PICRUSt predictions of nitrate reduction and denitrification pathways could be less accurate, given all the possible transformation steps involved. Future research integrating omics approaches and direct measurements of biogeochemical rates is needed to reveal actual biocrust microbial functions (e.g., Hungate et al., 2015).

Projected climatic changes in temperature and precipitation in the study site may directly or indirectly influence BSC communities and their ecosystem functions. Because biocrusts contribute to a large fraction of terrestrial biological N fixation and drive dryland emissions of reactive nitrogen (NO and HONO; Weber et al., 2015; Belnap et al., 2016; Barger et al., 2016; Chen et al., 2020), future research employing multi-omics and direct measurements of N metabolic rates will be required to fully delineate N cycling-related functional shifts in BSC communities with climatic change.

## Conclusion

Biological soil crusts are increasingly acknowledged as providing vital functions in drylands; however, the ecology of BSCs associated with colder, more mesic drylands in the Intermountain West is unknown. Using high-throughput sequencing, our study is the first to identify bacterial community shifts corresponding to changes in climate, and shrubs canopies within a rolling BSC landscape in cold steppe ecosystems. Findings from this study suggest that climate change as currently projected (warmer, snow-to-rain transition) could result in shifts of BSC community diversity and composition, including decreasing α diversity, replacement of putative rhizobial N_2_-fixers associated with shrub canopies by cyanobacterial N_2_-fixers in intershrub spaces, and increasing BSC nitrogenase activity. These alterations could have important ecosystem implications, as N fixation rate could affect ecosystem N budgets and soil nutrient availability and stability. Overall, our work provides an important addition to the literature of biocrust bacterial communities in sagebrush ecosystems, and serves as a baseline for future search toward a more mechanistic and predictive understanding of links between biocrust microbial community and ecosystem services in response to climate changes.

## Data Availability Statement

The datasets presented in this study can be found in online repositories. The names of the repository/repositories and accession number(s) can be found at: https://www.ncbi.nlm.nih.gov/, BioProject# PRJNA633217.

## Author Contributions

KAL and TSM designed the study. SGS and KAL collected the field samples and performed biogeochemical analyses. RNL led DNA extraction and 16S amplicon sequencing. YY managed the sequencing data and submitted them to the NCBI SRA. KA and YY conducted bioinformatics and ecological analyses and interpreted the results. KA, YY, and KAL wrote the manuscript. All authors contributed to the article and approved the submitted version.

### Conflict of Interest

The authors declare that the research was conducted in the absence of any commercial or financial relationships that could be construed as a potential conflict of interest.
