# Supplementary material for: Biological Soil Crust Bacterial Communities Vary Along Climatic and Shrub Cover Gradients Within a Sagebrush Steppe Ecosystem
Source: Front Microbiol. 2021 May 6;12:569791. doi: 10.3389/fmicb.2021.569791 (PMC8134670; doi:10.3389/fmicb.2021.569791)
Supplement: Supplementary file 1 [file Data_Sheet_1.PDF]

SUPPLEMENTARY MATERIAL

Biological soil crust bacterial communities vary along climatic and shrub cover gradients within a sagebrush steppe ecosystem

Contents

1 Experimental Design **1**

2 Software **2**

3 Environmental Analyses and Summaries **3**
3.1 Split Plot Analyses 3
3.2 Quantitative Summaries for Elevation, Grazing, and Shrub Levels 5
3.2.1 Summaries for Elevational Levels 5
3.2.2 Summaries for Grazing in Elevational Levels 6
3.2.3 Summaries for Shrub in Elevational Levels 8

4 Biocrust Community Analyses **10**
4.1 Multivariate Hypothesis Testing 10
4.1.1 Elevation 10
4.1.2 Grazing 10
4.1.3 Shrub 11
4.1.4 Summary 11
4.2 Ordination 12
4.2.1 Vector and Factor Fitting 13

5 Analysis of Taxa **15**
5.1 Taxa Summaries 15
5.2 Indicator Species Analysis 20
5.2.1 Elevation 21
5.2.2 Shrub 24
5.3 Nitrogen-cycling Traits Analysis 29

1 Experimental Design

Biological soil crust (BSC) samples were obtained within a split-split plot framework (shrub/intershrub within grazing/ungrazing within elevation) with blocking at two dates, August 2014 and October 2014 (Figure S1). Note that at each grazing level [grazing (G) or ungrazing (U)] replicate five shrub (S) and intershrub (I) samples were paired. Because of resource limitations, however, S and I observations were amalgamated into single samples within grazing levels, within elevation levels (Figure S1).

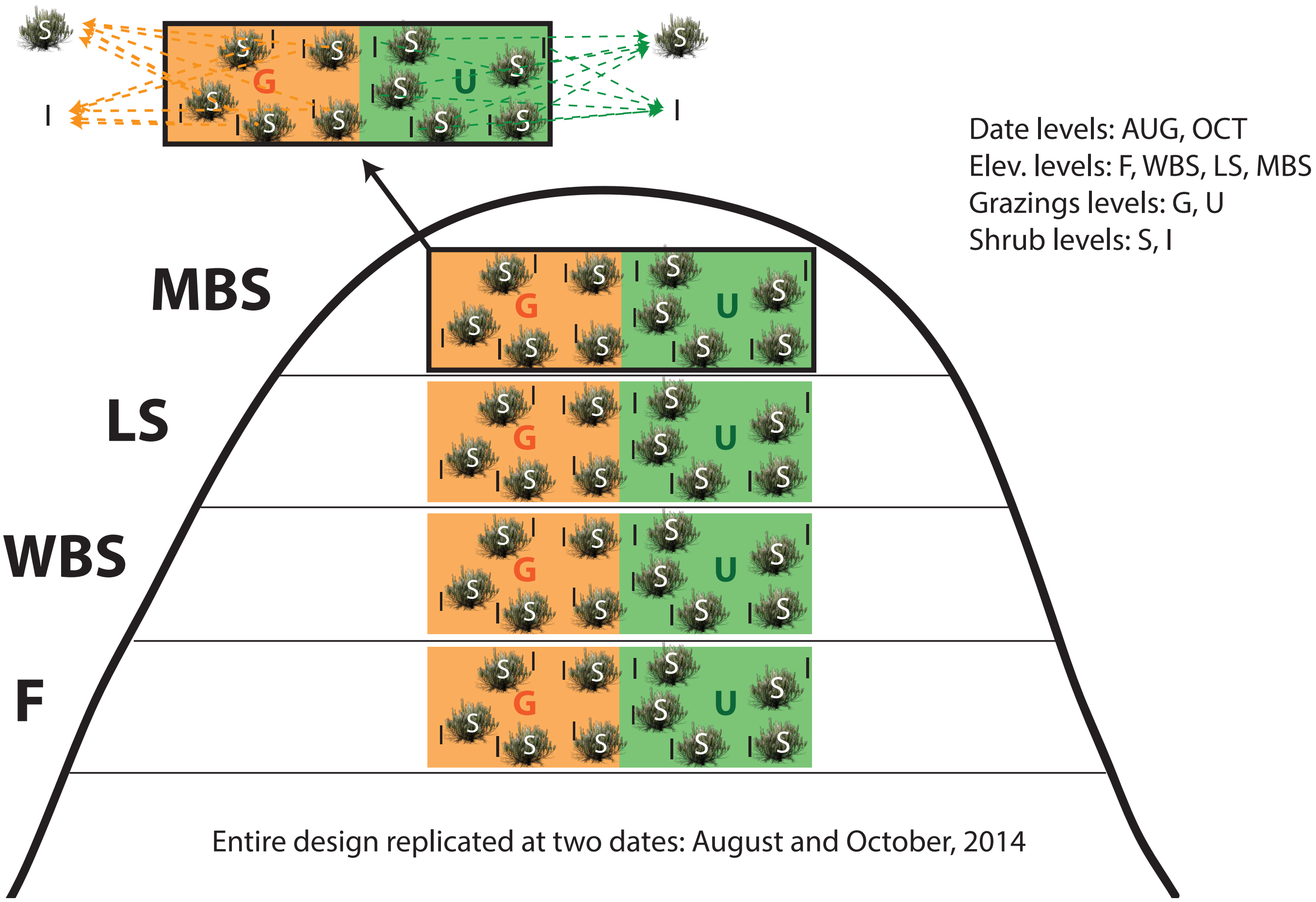

Figure S1. Experimental/sampling design for the study.

2 Software

All analyses we performed within the R statistical environment (R Core Team, 2018). We relied heavily on functions in the packages *vegan* (Oksanen *et al.*, 2018), *labdsv* (Roberts, 2016), *asbio* (Aho, 2018), *lme4* (Bates *et al.*, 2015), and *lmerTest* (Kuznetsova *et al.*, 2017).

3 Environmental Analyses and Summaries

3.1 Split Plot Analyses

Table S1. *P*-values for split plot analyses of biocrust and soil biogeochemical response variables. Largest replicated experimental factor with multiple levels (first column with a *P*-value) used a blocking factor in split plot frameworks because the resulting factors were unreplicated. For instance dates (Aug and Oct) for the NH_4 response variable. Blocking factors were treated as random effect variables. All lower stratum (nested) factors were treated as fixed effects. A check in the ”No conv.” column indicates indicates a failure in model convergence at specified tolerances. A check in the ”Sing.” column indicates that the model had a singular fit, and is likely overfitted (see Bates *et al.*, 2015)

	Date	Elev	Grazing	Shrub	E × G	E × S	G × S	E × G × S	No conv.	Sing.
Grav. water content	0.00034	1.6×10^{-5}	0.09	0.014	0.0062	0.076	0.74	0.99		
Biocrust cover		0.013	0.83	1×10^{-7}	0.055		0.41			
Bare ground cover		0.14	0.79	2.3×10^{-6}	0.26		0.49			✓
Vegetation cover		0.035	0.79	0.74	1		0.08			✓
Chlorophyll a		1	0.41	0.1	1		0.094			✓
NH_4	0.2	0.024	0.43	0.22	0.35	0.35	0.22	0.1		✓
NO_3	1	0.014	0.0025	0.001	0.011	1.9×10^{-5}	0.53	0.99		✓
Pot. net mineralization	1	0.0039	0.013	0.31	4.1×10^{-6}	0.61	0.33	0.054	✓	✓
Pot. net nitrification	0.047	0.009	0.22	0.23	0.094	0.00015	0.99	0.71	✓	
PO_4	1	0.035	2.6×10^{-13}	0.0003	8.4×10^{-22}	0.002	0.67	0.96		✓
pH	0.14	0.0049	0.98	0.75	0.24	0.8	0.69	0.88		
EC	0.15	0.19	0.32	0.29	0.23	1	0.47	0.7		
Soil ^{15}N		0.013	0.23	0.0022	0.32		0.68			
Soil ^{13}C		0.0053	0.17	0.045	1		0.86			
Soil N		0.0053	0.32	0.022	0.07		0.87			
Soil C		0.003	0.4	0.015	0.64		0.86			
Biocrust ^{15}N		0.28	0.97	0.00018	0.56		0.44			
Biocrust ^{13}C		0.066	0.5	0.003	0.04		0.7			
Biocrust N		0.022	0.82	6.8×10^{-5}	0.81		0.54			
Biocrust C		0.091	0.92	3.4×10^{-5}	0.35		0.7			
Pot. C_2H_4 produced		0.61	0.35	0.18	0.63		0.68			
Pot. C_2H_4 rate		0.82	0.44	0.61	1		0.88			✓
Pot. cover-adj C_2H_4 rate		0.075	0.87	6.9×10^{-15}	5.4×10^{-6}		0.14			✓
Pot. N-fixation		0.82	0.44	0.61	1		0.88			✓
Pot. cover-adj N-fixation		0.072	0.86	3.2×10^{-6}	2.6×10^{-9}		0.39			
Act. C_2H_4 produced		1	0.96	0.75	1		0.9			✓
Act. C_2H_4 rate		1	0.97	1	1		1			✓
Act. cover-adj C_2H_4 rate		0.056	0.92	4.9×10^{-11}	0.003		0.2			
Act. N-fixation		1	0.97	1	1		1			✓
Act. cover-adj N-fixation		0.056	0.92	0.00089	1.5×10^{-5}		0.52			

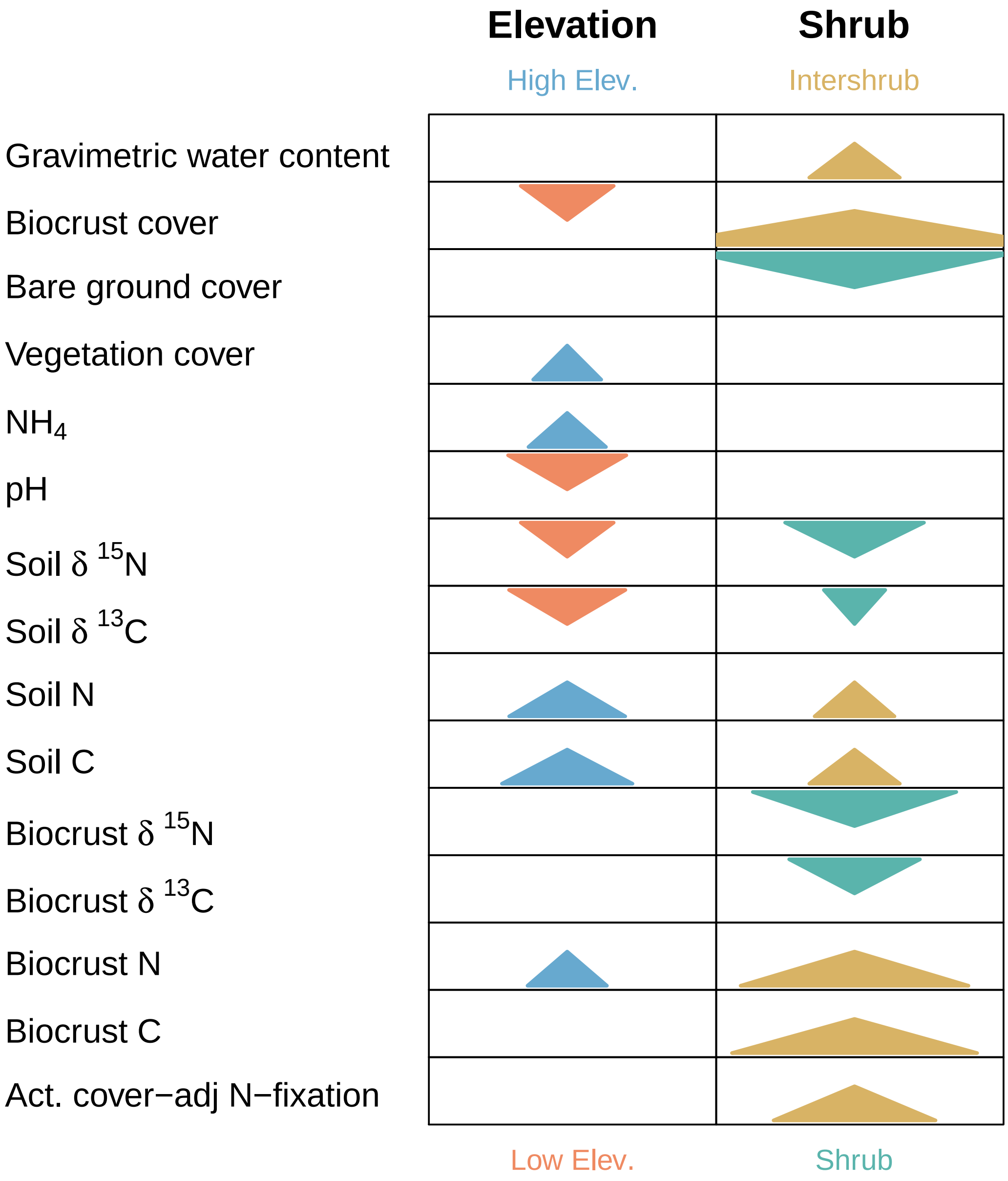

Figure S2. Graphical summary of significant analyses for biogeochemical response variables. Arrows indicate that main effects of statistically significance occurred in mixed models (see Table S1) along with an absence of significant interactions with other factors. Arrows point in direction of significant increase. Arrow width is scaled by the log of the inverse *p*-value. Thus, wider arrows constitute small *p*-values.

3.2 Quantitative Summaries for Elevation, Grazing, and Shrub Levels

3.2.1 Summaries for Elevational Levels

Table S2. Summary (n = number of date blocks, $\bar{x} \pm sd$), by elevational zone, for environmental characteristics. NAs are given for standard deviations when no replicates for an elevation level were obtained (e.g., measures were obtained for one date only). Shaded rows indicate that significant elevational main effects occurred and that significant interactions with elevation levels were absent.

	F	WBS	LS	MBS
Grav. water content	4, 0.05 ± 0.04	4, 0.08 ± 0.05	4, 0.12 ± 0.07	4, 0.25 ± 0.08
Biocrust cover	1, 58.1 ± NA	1, 59.55 ± NA	1, 35 ± NA	1, 1 ± NA
Bare ground cover	1, 29.3 ± NA	1, 27.45 ± NA	1, 28.35 ± NA	1, 65.15 ± NA
Vegetation cover	1, 12.6 ± NA	1, 13 ± NA	1, 36.65 ± NA	1, 33.85 ± NA
Chlorophyll a	1, 4.71 ± NA	1, 5.34 ± NA	1, 6.12 ± NA	1, 7.06 ± NA
NH_4	4, 0.87 ± 0.48	4, 1.3 ± 0.88	4, 1.43 ± 0.91	4, 3.78 ± 3.08
NO_3	4, 1.39 ± 1.12	4, 1 ± 0.46	4, 1.34 ± 0.91	4, 5.14 ± 2.49
Pot. net mineralization	4, 0.07 ± 0.09	4, 0.05 ± 0.03	4, 0.05 ± 0.08	4, 0.6 ± 0.36
Pot. net nitrification	4, 0.1 ± 0.19	4, 0.11 ± 0.17	4, 0.11 ± 0.11	4, 0.66 ± 0.52
PO_4	3, 6.24 ± 1.56	3, 10.35 ± 1.98	3, 10.34 ± 1.58	3, 10.91 ± 1.23
pH	3, 7.6 ± 0.76	3, 7.2 ± 0.15	3, 6.79 ± 0.37	3, 6 ± 0.22
EC	3, 269.99 ± 170.11	3, 190.19 ± 91.95	3, 136.3 ± 73.35	3, 114.77 ± 61.29
Soil ^{15}N	1, 8.56 ± NA	1, 7.59 ± NA	1, 7.16 ± NA	1, 4.74 ± NA
Soil ^{13}C	1, -21.58 ± NA	1, -25.22 ± NA	1, -25.87 ± NA	1, -26.3 ± NA
Soil N	1, 0.07 ± NA	1, 0.1 ± NA	1, 0.2 ± NA	1, 0.44 ± NA
Soil C	1, 0.67 ± NA	1, 1.18 ± NA	1, 2.56 ± NA	1, 5.65 ± NA
Biocrust ^{15}N	1, 5.52 ± NA	1, 4.64 ± NA	1, 3.7 ± NA	1, 3.69 ± NA
Biocrust ^{13}C	1, -22.88 ± NA	1, -25.73 ± NA	1, -26.56 ± NA	1, -26.8 ± NA
Biocrust N	1, 0.2 ± NA	1, 0.21 ± NA	1, 0.48 ± NA	1, 0.67 ± NA
Biocrust C	1, 2.78 ± NA	1, 2.66 ± NA	1, 7.4 ± NA	1, 9.99 ± NA
Pot. C_2H_4 produced	2, 4.43 ± 0.33	2, 4.36 ± 0.08	2, 4.37 ± 0.38	2, 4.24 ± 0.07
Pot. C_2H_4 rate	2, 44.82 ± 6.08	2, 43.88 ± 7.69	2, 44.54 ± 5.85	2, 42.62 ± 7.52
Pot. cover-adj C_2H_4 rate	2, 26.06 ± 3.56	2, 26.05 ± 4.44	2, 15.68 ± 2.17	2, 0.43 ± 0.08
Pot. N-fixation	2, 18.32 ± 2.49	2, 17.94 ± 3.14	2, 18.21 ± 2.39	2, 17.42 ± 3.07
Pot. cover-adj N-fixation	2, 10.69 ± 1.51	2, 10.68 ± 1.86	2, 6.4 ± 0.86	2, 0.17 ± 0.03
Act. C_2H_4 produced	2, 3.64 ± 0.68	2, 3.71 ± 0.81	2, 3.61 ± 0.64	2, 3.71 ± 0.82
Act. C_2H_4 rate	2, 37.91 ± 14.51	2, 38.29 ± 15.32	2, 37.86 ± 14.52	2, 38.23 ± 15.44
Act. cover-adj C_2H_4 rate	2, 22.02 ± 8.42	2, 22.81 ± 9.12	2, 13.26 ± 5.06	2, 0.38 ± 0.15
Act. N-fixation	2, 15.5 ± 5.93	2, 15.65 ± 6.26	2, 15.48 ± 5.94	2, 15.63 ± 6.31
Act. cover-adj N-fixation	2, 9 ± 3.44	2, 9.32 ± 3.73	2, 5.42 ± 2.07	2, 0.16 ± 0.06

3.2.2 Summaries for Grazing in Elevational Levels

Table S3. Overall summary for environmental characteristics (n = number of independent observations, $\bar{x} \pm sd$), within grazing levels. For grazing there were no cases in which grazing main effects were significant and significant interactions with grazing levels were absent.

	Grazed	Ungrazed
Grav. water content	16, 0.13 ± 0.12	16, 0.1 ± 0.08
Biocrust cover	4, 37.92 ± 31.02	4, 38.9 ± 32.5
Bare ground cover	4, 38.62 ± 29.66	4, 36.5 ± 29.98
Vegetation cover	4, 23.45 ± 19.65	4, 24.6 ± 24.54
Chlorophyll a	4, 6.28 ± 5.74	4, 5.34 ± 4.56
NH_4	16, 1.61 ± 1.91	16, 1.95 ± 9.82
NO_3	16, 2.65 ± 3.43	16, 1.57 ± 1.83
Pot. net mineralization	16, 0.22 ± 0.44	16, 0.12 ± 0.28
Pot. net nitrification	16, 0.26 ± 0.44	16, 0.16 ± 0.29
PO_4	12, 11.88 ± 7.91	12, 7.04 ± 5.3
pH	12, 6.9 ± 0.8	12, 6.97 ± 0.78
EC	12, 204.83 ± 235.55	12, 159.06 ± 125.22
Soil ^{15}N	4, 6.78 ± 1.45	4, 7.24 ± 1.97
Soil ^{13}C	4, -24.95 ± 1.96	4, -24.53 ± 2.62
Soil N	4, 0.22 ± 0.17	4, 0.19 ± 0.14
Soil C	4, 2.64 ± 2.28	4, 2.38 ± 2.12
Biocrust ^{15}N	4, 4.4 ± 1.78	4, 4.38 ± 2.05
Biocrust ^{13}C	4, -25.71 ± 1.55	4, -25.28 ± 2.52
Biocrust N	4, 0.4 ± 0.28	4, 0.38 ± 0.32
Biocrust C	4, 5.77 ± 4.97	4, 5.64 ± 5.83
Pot. C_2H_4 produced	8, 4.32 ± 0.33	8, 4.38 ± 0.31
Pot. C_2H_4 rate	8, 43.62 ± 5.42	8, 44.31 ± 5.84
Pot. cover-adj C_2H_4 rate	8, 16.69 ± 13.7	8, 17.42 ± 14.8
Pot. N-fixation	8, 17.83 ± 2.22	8, 18.11 ± 2.39
Pot. cover-adj N-fixation	8, 6.83 ± 4.79	8, 7.14 ± 5.57
Act. C_2H_4 produced	8, 3.67 ± 0.54	8, 3.66 ± 0.55
Act. C_2H_4 rate	8, 38.11 ± 10.64	8, 38.04 ± 10.65
Act. cover-adj C_2H_4 rate	8, 14.45 ± 12.83	8, 14.78 ± 13.39
Act. N-fixation	8, 15.58 ± 4.35	8, 15.55 ± 4.36
Act. cover-adj N-fixation	8, 5.91 ± 4.35	8, 6.04 ± 4.87

Table S4. Summary for environmental characteristics (n = number of independent observations, $\bar{x} \pm sd$), for grazing levels within elevation levels.

	F_G	F_U	WBS_G	WBS_U	LS_G	LS_U	MBS_G	MBS_U
Grav. water content	4, 0.05 ± 0.04	4, 0.04 ± 0.03	4, 0.07 ± 0.06	4, 0.08 ± 0.05	4, 0.11 ± 0.06	4, 0.13 ± 0.08	4, 0.29 ± 0.1	4, 0.2 ± 0.08
Biocrust cover	1, 46.4 ± 27.84	1, 69.8 ± 14.2	1, 61.6 ± 20.96	1, 57.5 ± 30.58	1, 42.7 ± 26.63	1, 27.3 ± 15.79	1, 1 ± 1.76	1, 1 ± 1.15
Bare ground cover	1, 39.3 ± 30.8	1, 19.3 ± 17.7	1, 23.7 ± 22.3	1, 31.2 ± 31.3	1, 22.3 ± 19.6	1, 34.4 ± 26.5	1, 69.2 ± 19.9	1, 61.1 ± 29.4
Vegetation cover	1, 14.3 ± 7.6	1, 10.9 ± 7.1	1, 14.7 ± 7.4	1, 11.3 ± 4.7	1, 35 ± 27.9	1, 38.3 ± 29.2	1, 29.8 ± 20.3	1, 37.9 ± 29.6
Chlorophyll a	1, 4.5 ± 3.68	1, 4.91 ± 3.87	1, 5.78 ± 4.81	1, 4.9 ± 3.3	1, 6.43 ± 5.71	1, 5.82 ± 6.6	1, 8.42 ± 8.04	1, 5.7 ± 4.44
NH_4	4, 0.94 ± 0.87	4, 0.8 ± 0.98	4, 1.47 ± 1.96	4, 1.13 ± 0.97	4, 1.55 ± 1.29	4, 1.31 ± 1.04	4, 2.48 ± 2.72	4, 5.44 ± 21.81
NO_3	4, 1.69 ± 2.06	4, 1.08 ± 1.31	4, 1.17 ± 0.9	4, 0.84 ± 0.41	4, 1.39 ± 1.18	4, 1.29 ± 1.17	4, 6.35 ± 4.77	4, 3.57 ± 2.74
Pot. net mineralization	4, 0.07 ± 0.15	4, 0.07 ± 0.13	4, 0.03 ± 0.12	4, 0.08 ± 0.17	4, 0.04 ± 0.14	4, 0.06 ± 0.15	4, 0.74 ± 0.6	4, 0.34 ± 0.51
Pot. net nitrification	4, 0.1 ± 0.21	4, 0.1 ± 0.24	4, 0.09 ± 0.15	4, 0.13 ± 0.28	4, 0.1 ± 0.17	4, 0.11 ± 0.14	4, 0.74 ± 0.62	4, 0.34 ± 0.42
PO_4	3, 6.46 ± 3.6	3, 6.01 ± 4.47	3, 12.8 ± 7.83	3, 7.9 ± 5.71	3, 9.46 ± 5.89	3, 11.22 ± 3.72	3, 18.79 ± 7.84	3, 3.04 ± 3.53
pH	3, 7.45 ± 0.89	3, 7.75 ± 0.73	3, 7.37 ± 0.25	3, 7.04 ± 0.5	3, 6.85 ± 0.45	3, 6.73 ± 0.27	3, 5.96 ± 0.3	3, 6.02 ± 0.41
EC	3, 388 ± 391	3, 152 ± 107	3, 196 ± 131	3, 185 ± 178	3, 135 ± 62	3, 138 ± 70	3, 101 ± 53	3, 163 ± 121
Soil ^{15}N	1, 7.9 ± 1.14	1, 9.21 ± 1.55	1, 7.41 ± 0.93	1, 7.78 ± 0.88	1, 7.04 ± 0.66	1, 7.27 ± 1.17	1, 4.79 ± 0.45	1, 4.69 ± 0.62
Soil ^{13}C	1, -22.2 ± 2	1, -20.9 ± 3	1, -25.2 ± 0.6	1, -25.2 ± 0.6	1, -25.9 ± 0.6	1, -25.8 ± 0.6	1, -26.4 ± 0.3	1, -26.2 ± 0.7
Soil N	1, 0.07 ± 0.02	1, 0.07 ± 0.02	1, 0.11 ± 0.04	1, 0.1 ± 0.03	1, 0.2 ± 0.06	1, 0.19 ± 0.1	1, 0.49 ± 0.05	1, 0.39 ± 0.08
Soil C	1, 0.67 ± 0.22	1, 0.67 ± 0.34	1, 1.25 ± 0.6	1, 1.1 ± 0.5	1, 2.49 ± 1.15	1, 2.63 ± 1.85	1, 6.16 ± 0.72	1, 5.14 ± 1.41
Biocrust ^{15}N	1, 5.08 ± 2.25	1, 5.96 ± 2.78	1, 4.84 ± 1.6	1, 4.44 ± 1.31	1, 3.42 ± 1.93	1, 3.98 ± 0.87	1, 4.25 ± 0.76	1, 3.13 ± 1.83
Biocrust ^{13}C	1, -23.9 ± 1.7	1, -21.9 ± 2.7	1, -25.9 ± 0.6	1, -25.6 ± 0.6	1, -26.3 ± 1.2	1, -26.8 ± 0.5	1, -26.8 ± 0.6	1, -26.8 ± 1
Biocrust N	1, 0.19 ± 0.14	1, 0.21 ± 0.13	1, 0.23 ± 0.15	1, 0.18 ± 0.08	1, 0.55 ± 0.33	1, 0.42 ± 0.24	1, 0.61 ± 0.14	1, 0.72 ± 0.39
Biocrust C	1, 2.61 ± 2.19	1, 2.94 ± 2.5	1, 3.07 ± 2.64	1, 2.25 ± 1.11	1, 8.92 ± 7.24	1, 5.88 ± 4.6	1, 8.49 ± 2.14	1, 11.49 ± 7.73
Pot. C_2H_4 produced	2, 4.39 ± 0.35	2, 4.47 ± 0.44	2, 4.3 ± 0.14	2, 4.42 ± 0.2	2, 4.43 ± 0.51	2, 4.32 ± 0.34	2, 4.15 ± 0.12	2, 4.32 ± 0.15
Pot. C_2H_4 rate	2, 44 ± 3.6	2, 45.7 ± 7.6	2, 43.3 ± 4.9	2, 44.5 ± 6.5	2, 45.2 ± 6.5	2, 43.9 ± 3.8	2, 42 ± 6.1	2, 43.2 ± 4.8
Pot. cover-adj C_2H_4 rate	2, 20.3 ± 11.9	2, 31.8 ± 7.8	2, 26.6 ± 9.3	2, 25.5 ± 13.5	2, 19.4 ± 12.2	2, 11.9 ± 6.7	2, 0.4 ± 0.7	2, 0.4 ± 0.5
Pot. N-fixation	2, 18 ± 1.5	2, 18.7 ± 3.1	2, 17.7 ± 2	2, 18.2 ± 2.7	2, 18.5 ± 2.6	2, 17.9 ± 1.5	2, 17.2 ± 2.5	2, 17.7 ± 2
Pot. cover-adj N-fixation	2, 8.35 ± 3.27	2, 13.03 ± 2.71	2, 10.89 ± 2.58	2, 10.47 ± 3.67	2, 7.89 ± 3.23	2, 4.9 ± 1.85	2, 0.17 ± 0.18	2, 0.18 ± 0.13
Act. C_2H_4 produced	2, 3.7 ± 0.6	2, 3.6 ± 0.5	2, 3.7 ± 0.6	2, 3.7 ± 0.6	2, 3.6 ± 0.5	2, 3.6 ± 0.5	2, 3.7 ± 0.6	2, 3.7 ± 0.6
Act. C_2H_4 rate	2, 37.9 ± 10.5	2, 37.9 ± 10.5	2, 38.4 ± 11.1	2, 38.2 ± 11.1	2, 37.9 ± 10.4	2, 37.8 ± 10.6	2, 38.3 ± 11.3	2, 38.2 ± 11.1
Act. cover-adj C_2H_4 rate	2, 17.61 ± 11.73	2, 26.44 ± 9.11	2, 23.62 ± 10.62	2, 21.99 ± 13.45	2, 16.19 ± 11.11	2, 10.33 ± 6.69	2, 0.38 ± 0.69	2, 0.38 ± 0.46
Act. N-fixation	2, 15.5 ± 4.31	2, 15.49 ± 4.3	2, 15.68 ± 4.53	2, 15.63 ± 4.56	2, 15.49 ± 4.27	2, 15.46 ± 4.35	2, 15.64 ± 4.61	2, 15.62 ± 4.55
Act. cover-adj N-fixation	2, 7.2 ± 2.95	2, 10.81 ± 3.19	2, 9.66 ± 3.23	2, 8.99 ± 3.52	2, 6.62 ± 2.78	2, 4.22 ± 1.7	2, 0.16 ± 0.14	2, 0.16 ± 0.1

3.2.3 Summaries for Shrub in Elevational Levels

Table S5. Overall summary for environmental characteristics (n = number of independent observations, $\bar{x} \pm sd$), within shrub levels. Shaded rows indicate that significant shrub main effects occurred and that significant interactions with shrub levels were absent.

	Intershrub	Shrub
Grav. water content	310, 0.12 ± 0.1	310, 0.11 ± 0.1
Biocrust cover	80, 50.2 ± 35.27	80, 26.62 ± 22.14
Bare ground cover	80, 25.05 ± 30.66	80, 50.08 ± 22.8
Vegetation cover	80, 24.75 ± 23.77	80, 23.3 ± 20.57
Chlorophyll a	80, 4.87 ± 5.14	80, 6.75 ± 5.09
NH_4	310, 1.41 ± 1.68	310, 2.14 ± 9.7
NO_3	310, 1.83 ± 2.22	310, 2.43 ± 3.3
Pot. net mineralization	310, 0.18 ± 0.38	310, 0.16 ± 0.37
Pot. net nitrification	310, 0.2 ± 0.32	310, 0.22 ± 0.43
PO_4	240, 10.68 ± 7.53	240, 8.24 ± 6.54
pH	230, 6.93 ± 0.78	230, 6.94 ± 0.8
EC	230, 192.01 ± 175.03	230, 173.87 ± 207.75
Soil ^{15}N	80, 6.68 ± 1.71	80, 7.34 ± 1.72
Soil ^{13}C	80, -25.05 ± 2.2	80, -24.43 ± 2.4
Soil N	80, 0.22 ± 0.16	80, 0.19 ± 0.16
Soil C	80, 2.79 ± 2.24	80, 2.24 ± 2.13
Biocrust ^{15}N	80, 3.67 ± 1.94	80, 5.11 ± 1.6
Biocrust ^{13}C	80, -25.93 ± 2.08	80, -25.06 ± 2.04
Biocrust N	80, 0.49 ± 0.33	80, 0.29 ± 0.22
Biocrust C	80, 7.67 ± 6.4	80, 3.74 ± 3.14
Pot. C_2H_4 produced	160, 4.32 ± 0.34	160, 4.38 ± 0.3
Pot. C_2H_4 rate	160, 43.74 ± 5.53	160, 44.19 ± 5.75
Pot. cover-adj C_2H_4 rate	160, 22.16 ± 15.87	160, 11.95 ± 10.12
Pot. N-fixation	160, 17.88 ± 2.26	160, 18.07 ± 2.35
Pot. cover-adj N-fixation	160, 7.9 ± 5.44	160, 6.07 ± 4.76
Act. C_2H_4 produced	160, 3.65 ± 0.53	160, 3.68 ± 0.57
Act. C_2H_4 rate	160, 38.07 ± 10.62	160, 38.07 ± 10.67
Act. cover-adj C_2H_4 rate	160, 19.11 ± 14.83	160, 10.12 ± 9.12
Act. N-fixation	160, 15.56 ± 4.34	160, 15.56 ± 4.36
Act. cover-adj N-fixation	160, 6.64 ± 4.59	160, 5.31 ± 4.55

Table S6. Summary for environmental characteristics (n = number of independent observations, $\bar{x} \pm sd$), for shrub levels within elevation levels. For simplicity, grazed and ungrazed levels are ignored.

	F_S	F_I	WBS_S	WBS_I	LS_S	LS_I	MBS_S	MBS_I
Grav. water content	40, 0.05 ± 0.04	40, 0.05 ± 0.04	40, 0.09 ± 0.06	40, 0.07 ± 0.05	40, 0.12 ± 0.07	40, 0.11 ± 0.07	35, 0.25 ± 0.11	35, 0.25 ± 0.1
Biocrust cover	10, 74.6 ± 8.78	10, 41.6 ± 24.46	10, 81.7 ± 6.13	10, 37.4 ± 15.99	10, 42.5 ± 29.37	10, 27.5 ± 10.12	10, 2 ± 1.49	10, 0 ± 0
Bare ground cover	10, 8.6 ± 7.2	10, 50 ± 21.9	10, 5.7 ± 5.5	10, 49.2 ± 20.7	10, 17.9 ± 17.2	10, 38.8 ± 25.1	10, 68 ± 29.5	10, 62.3 ± 20.2
Vegetation cover	10, 16.8 ± 3.9	10, 8.4 ± 7.7	10, 12.6 ± 6.5	10, 13.4 ± 6.3	10, 39.6 ± 31.5	10, 33.7 ± 24.9	10, 30 ± 29.8	10, 37.7 ± 20.2
Chlorophyll a	10, 6.11 ± 4.57	10, 3.3 ± 1.82	10, 5.73 ± 4.89	10, 4.96 ± 3.18	10, 4.53 ± 5.71	10, 7.72 ± 6.18	10, 3.11 ± 5.56	10, 11.01 ± 4.79
NH_4	40, 0.84 ± 0.86	40, 0.91 ± 1	40, 1.32 ± 1.05	40, 1.28 ± 1.93	40, 1.28 ± 1	40, 1.58 ± 1.31	35, 2.32 ± 2.86	35, 5.17 ± 20.16
NO_3	40, 1.17 ± 1.54	40, 1.61 ± 1.91	40, 1.07 ± 0.72	40, 0.94 ± 0.71	40, 1.39 ± 1.4	40, 1.29 ± 0.89	35, 3.96 ± 3.27	35, 6.35 ± 4.77
Pot. net mineralization	40, 0.09 ± 0.17	40, 0.05 ± 0.09	40, 0.09 ± 0.17	40, 0.01 ± 0.12	40, 0.04 ± 0.16	40, 0.06 ± 0.12	35, 0.56 ± 0.62	35, 0.57 ± 0.58
Pot. net nitrification	40, 0.12 ± 0.25	40, 0.08 ± 0.19	40, 0.16 ± 0.26	40, 0.06 ± 0.17	40, 0.09 ± 0.13	40, 0.12 ± 0.17	35, 0.46 ± 0.44	35, 0.68 ± 0.67
PO_4	30, 6.98 ± 4.62	30, 5.49 ± 3.26	30, 13.54 ± 7.86	30, 7.15 ± 4.84	30, 11.55 ± 4.87	30, 9.14 ± 4.84	30, 10.65 ± 10.14	30, 11.18 ± 9.96
pH	30, 7.62 ± 0.85	30, 7.58 ± 0.81	30, 7.18 ± 0.39	30, 7.23 ± 0.47	30, 6.75 ± 0.33	30, 6.83 ± 0.42	25, 6 ± 0.3	25, 5.97 ± 0.39
EC	30, 282 ± 268	30, 258 ± 348	30, 201 ± 147	30, 180 ± 165	30, 145 ± 65	30, 128 ± 67	25, 130 ± 103	25, 121 ± 79
Soil ^{15}N	10, 8.26 ± 1.58	10, 8.86 ± 1.4	10, 7.17 ± 1	10, 8.01 ± 0.56	10, 6.68 ± 1.02	10, 7.63 ± 0.55	10, 4.61 ± 0.59	10, 4.87 ± 0.46
Soil ^{13}C	10, -22.1 ± 2.5	10, -21.1 ± 2.6	10, -25.5 ± 0.5	10, -24.9 ± 0.5	10, -26.2 ± 0.6	10, -25.5 ± 0.3	10, -26.4 ± 0.5	10, -26.2 ± 0.5
Soil N	10, 0.08 ± 0.02	10, 0.06 ± 0.02	10, 0.12 ± 0.04	10, 0.09 ± 0.02	10, 0.23 ± 0.1	10, 0.16 ± 0.03	10, 0.44 ± 0.09	10, 0.44 ± 0.08
Soil C	10, 0.79 ± 0.26	10, 0.55 ± 0.26	10, 1.45 ± 0.61	10, 0.9 ± 0.29	10, 3.18 ± 1.91	10, 1.94 ± 0.51	10, 5.73 ± 1.18	10, 5.57 ± 1.3
Biocrust ^{15}N	10, 5.04 ± 2.86	10, 6 ± 2.13	10, 3.59 ± 0.68	10, 5.7 ± 1.2	10, 2.99 ± 1.34	10, 4.41 ± 1.32	10, 3.05 ± 1.68	10, 4.34 ± 0.94
Biocrust ^{13}C	10, -23.3 ± 2.5	10, -22.5 ± 2.5	10, -26.2 ± 0.4	10, -25.3 ± 0.4	10, -27.1 ± 0.7	10, -26 ± 0.8	10, -27.2 ± 0.8	10, -26.4 ± 0.6
Biocrust N	10, 0.28 ± 0.14	10, 0.12 ± 0.03	10, 0.29 ± 0.12	10, 0.12 ± 0.04	10, 0.61 ± 0.35	10, 0.36 ± 0.12	10, 0.76 ± 0.35	10, 0.57 ± 0.2
Biocrust C	10, 4.21 ± 2.53	10, 1.35 ± 0.42	10, 3.92 ± 2.18	10, 1.4 ± 0.57	10, 10.23 ± 7.58	10, 4.57 ± 1.83	10, 12.33 ± 6.96	10, 7.65 ± 2.95
Pot. C_2H_4 produced	20, 4.37 ± 0.5	20, 4.49 ± 0.25	20, 4.33 ± 0.14	20, 4.39 ± 0.21	20, 4.34 ± 0.4	20, 4.41 ± 0.47	20, 4.22 ± 0.17	20, 4.25 ± 0.16
Pot. C_2H_4 rate	20, 44.8 ± 7.1	20, 44.9 ± 4.8	20, 43.6 ± 5.3	20, 44.1 ± 6.3	20, 43.9 ± 4.1	20, 45.2 ± 6.3	20, 42.7 ± 5.5	20, 42.5 ± 5.6
Pot. cover-adj C_2H_4 rate	20, 33.4 ± 5.9	20, 18.8 ± 11.2	20, 35.6 ± 5	20, 16.5 ± 7.2	20, 18.8 ± 13.2	20, 12.5 ± 5.3	20, 0.8 ± 0.6	20, 0 ± 0
Pot. N-fixation	20, 18.3 ± 2.9	20, 18.3 ± 2	20, 17.8 ± 2.2	20, 18.1 ± 2.6	20, 17.9 ± 1.7	20, 18.5 ± 2.6	20, 17.4 ± 2.2	20, 17.4 ± 2.3
Pot. cover-adj N-fixation	20, 12.07 ± 2.82	20, 9.31 ± 4.2	20, 12.36 ± 0.9	20, 9 ± 3.67	20, 6.91 ± 3.55	20, 5.88 ± 2.32	20, 0.25 ± 0.16	20, 0.1 ± 0.1
Act. C_2H_4 produced	20, 3.6 ± 0.4	20, 3.7 ± 0.6	20, 3.7 ± 0.6	20, 3.7 ± 0.6	20, 3.6 ± 0.5	20, 3.6 ± 0.5	20, 3.7 ± 0.6	20, 3.7 ± 0.6
Act. C_2H_4 rate	20, 37.9 ± 10.5	20, 37.9 ± 10.6	20, 38.3 ± 11.1	20, 38.3 ± 11.1	20, 37.8 ± 10.5	20, 37.9 ± 10.6	20, 38.2 ± 11.2	20, 38.2 ± 11.2
Act. cover-adj C_2H_4 rate	20, 28.29 ± 8.49	20, 15.75 ± 10.33	20, 31.29 ± 9.37	20, 14.32 ± 7.47	20, 16.1 ± 12.08	20, 10.42 ± 4.83	20, 0.76 ± 0.62	20, 0 ± 0
Act. N-fixation	20, 15.51 ± 4.28	20, 15.48 ± 4.33	20, 15.66 ± 4.54	20, 15.65 ± 4.55	20, 15.46 ± 4.29	20, 15.5 ± 4.33	20, 15.63 ± 4.58	20, 15.63 ± 4.57
Act. cover-adj N-fixation	20, 9.94 ± 2.35	20, 8.07 ± 4.29	20, 10.57 ± 1.53	20, 8.08 ± 4.18	20, 5.84 ± 2.77	20, 5 ± 2.37	20, 0.21 ± 0.11	20, 0.1 ± 0.1

4 Biocrust Community Analyses

4.1 Multivariate Hypothesis Testing

We used PERMANOVA to test multivariate null hypotheses of no effect of elevation (whole plot), grazing (split plot) and shrub/intershrub on bacterial community OTU composition. Bray-Curtis dissimilarity (Bray & Curtis, 1957) was used as the underlying resemblance matrices in both PERMANOVA and ordinations (see below). The split-split plot design required three analytical components.

4.1.1 Elevation

First, to determine elevational (whole plot) effects, we obtained OTU multivariate centroids of whole plots in date blocks.

```
centroid.wp <- matrix(ncol = ncol(data), nrow = nlevels(interaction(elev, date)))
for(i in 1:ncol(centroid.wp)){
centroid.wp[,i] <- tapply(data[,i], paste(elev, date, sep="_"), mean)
}

# Define treatments to go with the centroids
temp <- tapply(data[,1], paste(elev, date, sep="_"), mean)
x <- noquote(strsplit(names(temp), "_"))

exp.wp <- matrix(ncol = 2, nrow = nrow(centroid.wp), data = unlist(x), byrow = T,
dimnames=list(1:nrow(centroid.wp), c("elev", "date")))
exp.wp <- data.frame(exp.wp)
```

The PERMANOVA for elevation (whole plot) effects was run on the eight observations (four elevations at two dates), blocking by date.

```
library(vegan)
# model --
wp <- adonis(centroid.wp ~ date + elev, data = exp.wp, method = 'bray')
```

4.1.2 Grazing

Second, to assess grazing effects, and the interaction of elevation and grazing, we obtained centroids for grazing levels in date in elevation as observational units.

```
centroid.sp <- matrix(ncol = ncol(data), nrow = nlevels(interaction(grazing, elev, date)))
for(i in 1:ncol(centroid.sp)){
centroid.sp[,i] <- tapply(data[,i], paste(grazing, elev, date, sep="_"), mean)
}
n <- tapply(data[,1], paste(grazing, elev, date, sep="_"), mean)
rownames(centroid.sp) <- names(n)

# Define treatments to go with the centroids
x <- noquote(strsplit(names(n), "_"))
exp.sp <- matrix(ncol = 3, nrow = nrow(centroid.sp), data = unlist(x), byrow = T,
dimnames=list(names(n), c("grazing", "elev", "date")))
exp.sp <- data.frame(exp.sp)
```

The code below uses the correction factor `paste(date, elev)` to obtain the correct (split plot) df and test statistics for other factors.

```
# model --
whole.plot <- with(exp.sp, paste(date, elev))
sp <- adonis(centroid.sp ~ whole.plot + elev * grazing,
       data = exp.sp, method = 'bray')
```

4.1.3 Shrub

Third, we used the correction factor `paste(date, elev, grazing)` to obtain the correct (split-split-plot) df and test statistics for other factors.

```
# model --
split.plot <- paste(date, elev, grazing)
ssp <- adonis(data ~ split.plot + PI + PI : grazing + PI : elev + PI : elev : grazing,
method = 'bray')
```

4.1.4 Summary

Below we denote the number of whole plot (elevation) levels as $a = 4$ with individual levels F, WBS, LS and MBS, the number of blocks (dates) as $b = 2$ with individual levels Aug and Oct, the number of split plot (grazing) levels as $c = 2$ with individual levels G and U, and the number of shrub/interspace levels as $d = 2$ with individual levels S and I (see Figure S1).

Note that the ANOVA model framework described in the preceding sections result in the correct degrees of freedom for a split-split-plot design (Table S7).

- At the whole-plot level we have:
  - $a-1 = 4-1 = 3$, degrees of freedom for elevation,
  - $b-1 = 2-1 = 1$ df for date, and
  - $ab-1 = 4\times 2-1 = 7$ error degrees of freedom.
- At the split-plot level we have:
  - $c-1 = 2-1 = 1$ degree of freedom for grazing,
  - $(a-1)(c-1) = 3(1) = 3$ degrees of freedom for the grazing $\times$ elevation interaction, and
  - $a(b-1)(c-1) = 4(1)(1) = 4$ error degrees of freedom.
- At the split-split-plot level we have:
  - $d-1 = 2-1 = 1$ degree of freedom for shrub,
  - $(d-1)(c-1) = (1)(1) = 1$ df for the shrub $\times$ grazing interaction,
  - $(d-1)(a-1) = (1)(3) = 3$ df for the shrub $\times$ elevation interaction,
  - $(d-1)(c-1)(a-1) = (1)(1)(3) = 3$ df for the shrub $\times$ grazing $\times$ elevation interaction, and
  - $(b-1)(ac)(d-1) = (1)(4)(2)(1) = 8$ error degrees of freedom.
  - The total degrees of freedom $(a\times b\times c\times d)-1 = (4)(2)(2)(2)-1 = 31$ are also correct.

See Aho (2016) for additional information.

The split-split-plot PERMANOVA test results are summarized in Table S7.

- At the whole-plot level:
  - **Date** was not significant.
  - **Elevation**, was strongly significant.
- At the split-plot level:
  - **Grazing** was not significant. The **grazing** × **elevation** interaction was not significant.
- At the split-split-plot level:
  - **Shrub/interspace** effects were strongly significant.
  - The **grazing** × **shrub interaction** was not significant.
  - The **elevation** × **shrub interaction** was not significant.
  - The **elevation** × **grazing** × **shrub interaction** was not significant.

Table S7. Summary of split-split-plot PERMANOVA for community composition

	df	*SS*	*MS*	F^*	*p*-value
**Whole-Plot**					
Date	1	0.055	0.055	2.273	0.148
Elevation	3	0.558	0.186	7.666	0.002
Whole-Plot Error	3	0.073	0.024		
**Split-plot**					
Grazing	1	0.049	0.049	1.09	0.347
Grazing × Elevation	3	0.177	0.059	1.317	0.261
Split-Plot Error	4	0.179	0.045		
**Split-Split-Plot**					
Shrub	1	0.323	0.323	3.666	0.009
Shrub × Grazing	1	0.053	0.053	0.6	0.812
Shrub × Elevation	3	0.277	0.092	1.047	0.421
Shrub × Grazing × Elevation	3	0.189	0.063	0.713	0.83
Split-Split-Plot Error	8	0.706	0.088		
Total	31				

4.2 Ordination

We used non-metric multidimensional scaling (NMDS) ordination (Legendre & Legendre, 2012) to depict relationships of samples in OTU space.

To decrease the propensity of biogeochemical characteristics or OTUs to influence the NMDS projection, we applied a Wisconsin double transformation (Bray & Curtis, 1957) to the biocrust samples by OTU data matrix.

The two-dimensional NMDS solution was inferentially strong (stress = 0.089). Patterns detected in the split-split plot PERMANOVA were shown with remarkable clarity in the ordination (Figure S3). The first axis follows the elevational gradient, from low to high elevation. The second axis depicts community differences in plant-interspace levels.

4.2.1 Vector and Factor Fitting

Correlations of environmental variables to the projection were computed using vector fitting for quantitative predictors and factor fitting for categorical predictors (Legendre & Legendre, 2012; Oksanen *et al.*, 2018).

A large number of quantitative soil biogeochemical predictors were significantly correlated with the projection (Figure S3, Table S8). Strongest correlates were soil δ^{15} N ($r^2 = 0.96$), soil %C ($r^2 = 0.93$), soil %N ($r^2 = 0.92$), pH ($r^2 = 0.92$), GWC ($r^2 = 0.84$), crust %N ($r^2 = 0.81$), and soil NH_4 ($r^2 = 0.79$) (Table S8). Reflecting PERMANOVA results, elevation was significantly correlated with the projection ($r^2 = 0.86$). Shrub/intershrub levels, however, were not significantly correlated with the NMDS projection.

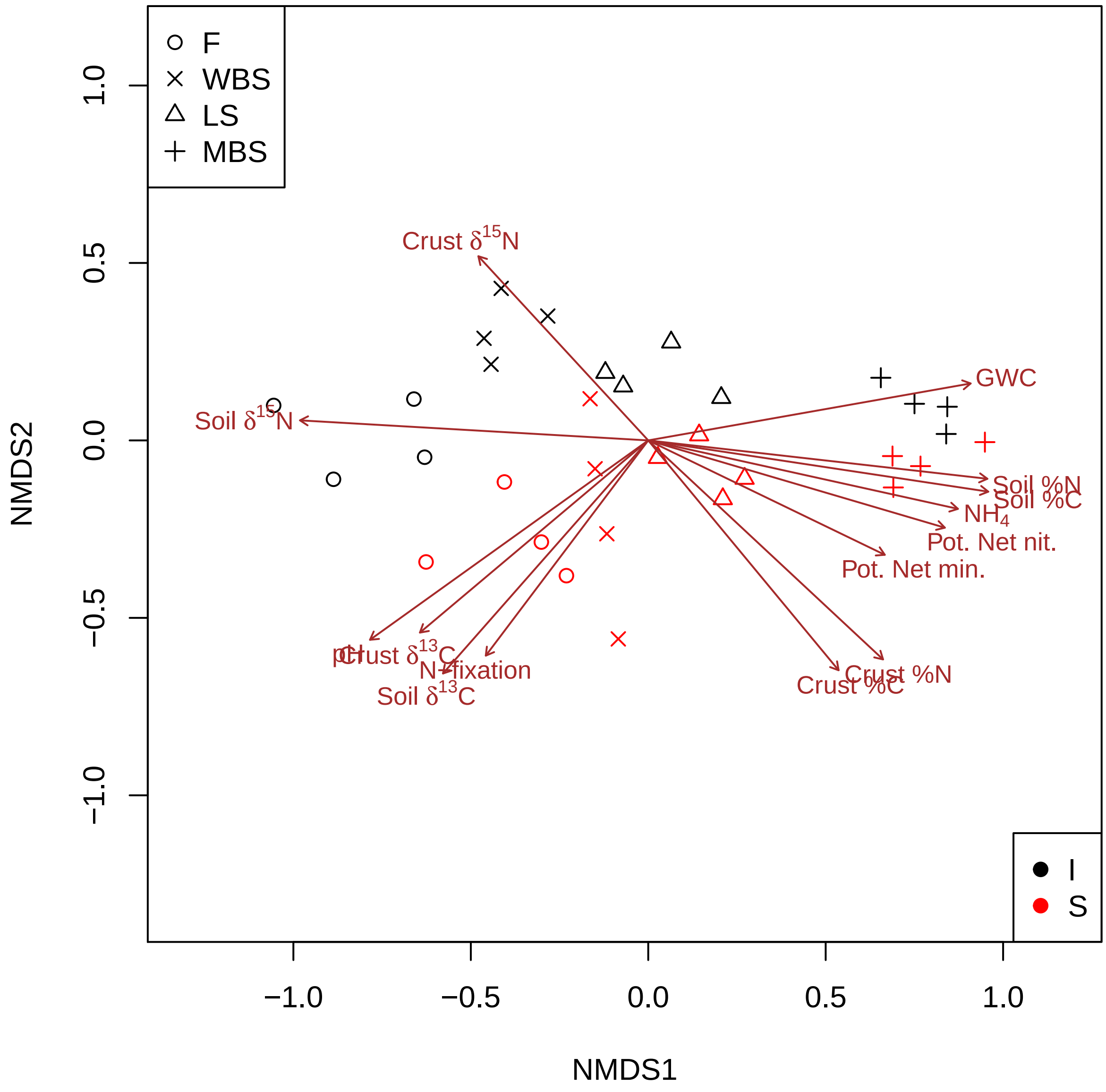

Figure S3. NMDS ordination of biocrust OTUs. Elevation and shrub/intershrub levels are shown. Environmental factors significantly correlating with the projection (p-values ≤ 0.05) are overlain.

Table S8. Correlations of environmental factors to the NMDS projection.

	r^2	p-value
**Vectors**		
GWC	0.844	0.001
Chl a	0.102	0.485
NH_4	0.792	0.001
NO_3	0.018	0.888
Potential net mineralization	0.543	0.004
Potential net nitrification	0.753	0.001
PO_4	0.111	0.479
pH	0.922	0.001
EC	0.090	0.536
Soil $\delta^{15}N$	0.958	0.001
Soil $\delta^{13}C$	0.759	0.001
Soil %N	0.917	0.001
Soil %C	0.931	0.001
Crust $\delta^{15}N$	0.494	0.014
Crust $\delta^{13}C$	0.701	0.002
Crust %N	0.811	0.001
Crust %C	0.701	0.001
Cover-adj. Pot. N-fixation	0.595	0.008
Cover-adj. Actual N-fixation	0.572	0.010
**Factors**		
Date	0.000	1.000
Elevation	0.861	0.001
Shrub	0.091	0.246
Grazing	0.006	0.885

5 Analysis of Taxa

5.1 Taxa Summaries

A summary of the overall number of distinct taxa observed within taxonomic hierarchies is shown in Table S9. Summaries of most abundance (highest read) phyla, classes, orders, families and genera are provided in Tables S10, S11, S12, S13, and S14, respectively.

Table S9. Number of distinct levels within taxonomic hierarchies. Unknown, uncultured, and unclassified designations are eliminated.

Kingdom	Phylum	Class	Order	Family	Genus
1	26	63	155	266	464

Table S10. Twenty most abundant phyla in BSC communities at all sites.

	Rank	Prop. of reads	Cum. prop. of reads
Cyanobacteria	1	0.1102	0.1102
Actinobacteria	2	0.0855	0.1957
WS2	3	0.0697	0.2654
Acidobacteria	4	0.0627	0.3281
Nitrospirae	5	0.0620	0.3901
Armatimonadetes	6	0.0611	0.4511
Chloroflexi	7	0.0609	0.5120
Proteobacteria	8	0.0572	0.5692
Bacteroidetes	9	0.0523	0.6215
Gemmatimonadetes	10	0.0507	0.6722
Planctomycetes	11	0.0454	0.7176
Verrucomicrobia	12	0.0443	0.7618
Firmicutes	13	0.0388	0.8006
FBP	14	0.0341	0.8347
Fibrobacteres	15	0.0338	0.8684
Deinococcus.Thermus	16	0.0316	0.9001
BRC1	17	0.0262	0.9263
Unknown/Unclassified	18	0.0163	0.9426
Patescibacteria	19	0.0154	0.9580
Entotheonellaeota	20	0.0129	0.9710

Table S11. Thirty most abundant classes in BSC communities at all sites.

	Rank	Prop. of reads	Cum. prop. of reads
Unknown/Unclassified	1	0.0927	0.0927
Actinobacteria	2	0.0452	0.1380
Oxyphotobacteria	3	0.0431	0.1811
Alphaproteobacteria	4	0.0425	0.2236
Rubrobacteria	5	0.0410	0.2645
KD4.96	6	0.0391	0.3037
Acidobacteriia	7	0.0368	0.3405
Phycisphaerae	8	0.0340	0.3745
Anaerolineae	9	0.0318	0.4064
TK10	10	0.0318	0.4381
Armatimonadia	11	0.0297	0.4678
Blastocatellia_Subgroup_4	12	0.0295	0.4973
Acidimicrobiia	13	0.0287	0.5260
Chloroflexia	14	0.0281	0.5540
WS2_cl	15	0.0266	0.5807
Nitrospira	16	0.0250	0.6057
Fimbriimonadia	17	0.0214	0.6270
Thermoleophilia	18	0.0207	0.6477
Gemmatimonadetes	19	0.0201	0.6678
Bacteroidia	20	0.0201	0.6879
Subgroup_6	21	0.0174	0.7053
Verrucomicrobiae	22	0.0169	0.7222
Bacilli	23	0.0165	0.7387
Subgroup_5	24	0.0149	0.7536
Gammaproteobacteria	25	0.0137	0.7673
Holophagae	26	0.0136	0.7809
Chthonomonadetes	27	0.0134	0.7942
Dehalococcoidia	28	0.0131	0.8073
FBP_cl	29	0.0130	0.8203
Fibrobacteria	30	0.0129	0.8332

Table S12. Thirty most abundant orders in BSC communities at all sites.

	Rank	Prop. of reads	Cum. prop. of reads
Unknown/Unclassified	1	0.1104	0.1104
Actinobacteria	2	0.0358	0.1462
Oxyphotobacteria	3	0.0326	0.1788
Alphaproteobacteria	4	0.0230	0.2018
Rubrobacteria	5	0.0230	0.2248
KD4.96	6	0.0210	0.2458
Acidobacteriia	7	0.0199	0.2657
Phycisphaerae	8	0.0193	0.2849
Anaerolineae	9	0.0191	0.3040
TK10	10	0.0191	0.3231
Armatimonadia	11	0.0188	0.3419
Blastocatellia_Subgroup_4	12	0.0183	0.3602
Acidimicrobiia	13	0.0178	0.3780
Chloroflexia	14	0.0177	0.3957
WS2_cl	15	0.0175	0.4132
Nitrospira	16	0.0165	0.4297
Fimbriimonadia	17	0.0164	0.4461
Thermoleophilia	18	0.0164	0.4625
Gemmatimonadetes	19	0.0157	0.4781
Bacteroidia	20	0.0156	0.4938
Subgroup_6	21	0.0148	0.5085
Verrucomicrobiae	22	0.0142	0.5227
Bacilli	23	0.0141	0.5368
Subgroup_5	24	0.0139	0.5506
Gammaproteobacteria	25	0.0127	0.5633
Holophagae	26	0.0125	0.5758
Chthonomonadetes	27	0.0119	0.5877
Dehalococcoidia	28	0.0119	0.5996
FBP_cl	29	0.0112	0.6108
Fibrobacteria	30	0.0110	0.6218

Table S13. Forty most abundant families in BSC communities at all sites.

	Rank	Prop. of reads	Cum. prop. of reads
Unknown/Unclassified	1	0.1334	0.1334
Labraceae	2	0.0343	0.1676
Iamiaceae	3	0.0232	0.1908
Kineosporiaceae	4	0.0188	0.2096
Caldilineaceae	5	0.0171	0.2267
Nakamurellaceae	6	0.0169	0.2436
Micrococcaceae	7	0.0155	0.2592
Bacillaceae	8	0.0149	0.2740
Ilumatobacteraceae	9	0.0148	0.2889
Propionibacteriaceae	10	0.0136	0.3024
Microcystaceae	11	0.0132	0.3157
Beijerinckiaceae	12	0.0127	0.3283
Geodermatophilaceae	13	0.0126	0.3409
Mycobacteriaceae	14	0.0122	0.3531
Sphingomonadaceae	15	0.0121	0.3652
Nostocaceae	16	0.0112	0.3764
Xanthobacteraceae	17	0.0111	0.3875
Micromonosporaceae	18	0.0110	0.3986
Intrasporangiaceae	19	0.0107	0.4093
Pseudonocardiaceae	20	0.0100	0.4193
Nocardioidaceae	21	0.0097	0.4290
Micropepsaceae	22	0.0096	0.4386
Cellulomonadaceae	23	0.0096	0.4482
Rhizobiales_Incertae_Sedis	24	0.0094	0.4576
Acetobacteraceae	25	0.0094	0.4670
Rhizobiaceae	26	0.0093	0.4764
Promicromonosporaceae	27	0.0091	0.4854
Microbacteriaceae	28	0.0090	0.4945
bacteriap25	29	0.0090	0.5034
Streptomycetaceae	30	0.0087	0.5121
Acidobacteriaceae_Subgroup_1	31	0.0086	0.5207
Solibacteraceae_Subgroup_3	32	0.0086	0.5293
TRA3.20	33	0.0086	0.5379
Rubrobacteriaceae	34	0.0086	0.5465
Streptosporangiaceae	35	0.0085	0.5550
Blastocatellaceae	36	0.0082	0.5632
KD4.96_fa	37	0.0082	0.5714
Rhodobacteraceae	38	0.0078	0.5792
Caulobacteraceae	39	0.0077	0.5869
Cryptosporangiaceae	40	0.0076	0.5945

Table S14. Sixty most abundant genera in BSC communities at all sites.

	Rank	Prop. of reads	Cum. prop. of reads
Unknown/Unclassified	1	0.1720	0.1720
Actinoplanes	2	0.0840	0.2560
Sphingomonas	3	0.0277	0.2837
Salinibacterium	4	0.0223	0.3060
Mesorhizobium	5	0.0211	0.3271
Arthrobacter	6	0.0201	0.3472
Streptomyces	7	0.0196	0.3668
Allorhizobium.Neorhizobium.Pararhizobium.Rhizobium	8	0.0189	0.3857
Clostridium_sensu_stricto_13	9	0.0157	0.4014
Erythrobacter	10	0.0144	0.4158
Pantoea	11	0.0127	0.4284
Labrys	12	0.0125	0.4409
CL500.29_marine_group	13	0.0118	0.4527
Bacillus	14	0.0111	0.4639
Novosphingobium	15	0.0111	0.4749
Methylobacterium	16	0.0106	0.4855
Microbacterium	17	0.0099	0.4954
Roseomonas	18	0.0096	0.5050
Rhodopseudomonas	19	0.0090	0.5140
Iamia	20	0.0084	0.5224
Myxococcus	21	0.0084	0.5309
Kineosporia	22	0.0082	0.5391
Herbaspirillum	23	0.0079	0.5470
Actinotalea	24	0.0076	0.5546
Caulobacter	25	0.0071	0.5617
Streptosporangium	26	0.0071	0.5688
Acidipila	27	0.0063	0.5752
Chelatococcus	28	0.0063	0.5815
Amycolatopsis	29	0.0062	0.5876
Nakamurella	30	0.0061	0.5938
Yonghaparkia	31	0.0059	0.5997
Microtetraspora	32	0.0055	0.6053
Cellulomonas	33	0.0053	0.6105
Rhodobacter	34	0.0051	0.6157
Modestobacter	35	0.0051	0.6207
Lysinibacillus	36	0.0049	0.6257
Kibdelosporangium	37	0.0049	0.6306
Curtobacterium	38	0.0048	0.6354
Acidiphilium	39	0.0048	0.6401
Dactylosporangium	40	0.0046	0.6448
Granulicella	41	0.0046	0.6494
Mycobacterium	42	0.0045	0.6539
Bosea	43	0.0044	0.6583
Eoetvoesia	44	0.0043	0.6626
Pseudonocardia	45	0.0042	0.6668
Phenylobacterium	46	0.0041	0.6710
OLB8	47	0.0039	0.6749
JGI_0001001.H03	48	0.0039	0.6788
Blastocatella	49	0.0038	0.6825
Marmoricola	50	0.0038	0.6863

5.2 Indicator Species Analysis

Indicator species analysis (ISA; Dufrene & Legendre, 1997) was used to test for association of taxa designations with elevation and shrub levels. All undefined taxa (i.e., "unclassified", "uncultured", "unknown") were eliminated from ISA analysis. Specific taxa were required to occur in two or more samples for inclusion in ISA analyses. *P*-values for tests of the null hypothesis that associations between taxa and categorical assignments were no better than random were based on permutation procedures using 1000 iterations. *All* taxa were included in graphical summaries of ISA

results to show the proportion of undefined OTUs at particular taxonomic hierarchies.

5.2.1 Elevation

Table S15 provides richness and diversity summaries for OTUs and Linnean taxonomic summaries within elevation levels. Table S16 summarizes significant ISA results across all elevation levels for each taxonomic hierarchy. Table S17 provides a summary of significant ISA results for each taxonomic hierarchy, for each elevational level. Table S18-S20 show indicator taxa, at various taxonomic level, across elevation zones.

Table S15. Richness and diversity summary for elevation levels. Except for β-diversity (which was measured as mean Bray-Curtis dissimilarity among samples), entries are $\bar{x} \pm$ SEM.

	F	WBS	LS	MBS
OTU richness	9897.88 ± 671.17	10894.88 ± 969.92	13919.88 ± 613.43	13611.75 ± 526.9
OTU $\alpha-$diversity	6.47 ± 0.12	6.77 ± 0.09	7.18 ± 0.07	7.3 ± 0.05
OTU $\beta-$diversity	0.48	0.37	0.39	0.46
No. Phylum	22.75 ± 0.25	23.12 ± 0.4	24 ± 0.27	23.5 ± 0.19
No. Class	52.38 ± 0.6	53.38 ± 1.19	57.38 ± 0.42	54.5 ± 0.57
No. Order	110.5 ± 2.43	111.62 ± 3.17	120.88 ± 1.57	118.75 ± 0.94
No. Family	228.25 ± 5.67	230.62 ± 6.39	248.5 ± 4.18	252.75 ± 3
No. Genus	360.25 ± 9.18	365 ± 12.77	401.12 ± 6.76	414.12 ± 3.85

Table S16. Overall significant ($\alpha = 0.05$) ISA results for elevations for the indicated taxonomic levels.

	Phylum	Class	Order	Family	Genus
No. of taxa considered	24	61	146	247	410
No. unadj. signif. indicators	14	33	66	109	169
No. FDR-adj. signif. indicators	11	24	42	80	116
Prop. of unadj. signif. indicators	0.5833	0.541	0.4521	0.4413	0.4122
Prop. signif. FDR-adj. indicators	0.4583	0.3934	0.2877	0.3239	0.2829

Table S17. Numbers of significant ($\alpha = 0.05$) ISA results for elevation for the indicated taxonomic and elevation levels. Semicolons separate significant unadjusted and FDR-adjusted results.

	F	WBS	LS	MBS
Phylum	3;2	0;0	7;6	4;3
Class	3;2	0;0	19;14	11;8
Order	10;5	1;0	25;13	30;24
Family	14;11	5;3	31;17	59;49
Genus	21;16	6;3	40;16	102;81

Table S18. Significant ($\alpha = 0.05$) ISA at the phylum, class, and order level for particular elevations (F = 1, WBS = 2, LS = 3, MBS = 4). Asterisked entries indicate significant (at $\alpha = 0.05$) indicators after adjustment for false discovery rate.

Phylum	lvl	Class	lvl	Order	lvl
Chloroflexi*	3	Acidobacteriia*	4	Acidobacteriales*	4
Cyanobacteria*	1	Actinobacteria	4	Anaerolineales*	3
Deinococcus-Thermus	1	Anaerolineae*	3	Bacillales*	4
Elusimicrobia*	3	Bacilli*	4	Bacteroidales*	4
Entotheonellaeota*	3	Chloroflexia*	3	BD2-11_terrestrial_group_or	3
FBP*	1	Chthonomonadetes*	3	Betaproteobacteriales*	4
Fibrobacteres*	3	Clostridia*	4	C0119	3
Firmicutes*	4	Dehalococcoidia*	3	Caldilineales*	3
Gemmatimonadetes*	4	Deinococci	1	Candidatus_Adlerbacteria	3
Nitrospirae*	3	Elusimicrobia*	3	Candidatus_Peribacteria	3
Omnitrophicaeota	3	Entotheonellia*	3	CHAB-XI-27*	1
Patescibacteria*	4	FBP_cl*	1	Chloroflexales*	3
Verrucomicrobia	4	Fibrobacteria*	3	Chthoniobacterales*	4
WS2*	3	Gammaproteobacteria*	4	Chthonomonadales*	3
.	.	Gemmatimonadetes*	4	Clostridiales*	4
.	.	Gracilibacteria	3	Corynebacteriales*	4
.	.	Holophagae	3	Cytophagales	1
.	.	Ktedonobacteria	3	Deinococcales	1
.	.	Melainabacteria	4	Desulfarculales	4
.	.	Microgenomatia	3	Desulfuromonadales*	4
.	.	Negativicutes*	4	Diplorickettsiales*	4
.	.	Nitrospira*	3	Elsterales*	4
.	.	Omnitrophicaeota_cl	3	Entotheonellales*	3
.	.	Oxyphotobacteria*	1	Euzebyales	1
.	.	Pla4_lineage*	3	FBP_or*	1
.	.	Rubrobacteria*	3	Fibrobacterales*	3
.	.	Saccharimonadia*	4	Frankiales*	4
.	.	Subgroup_17*	3	Gaiellales	4
.	.	Subgroup_25*	3	Gammaproteobacteria_Incertae_Sedis*	4
.	.	Thermoleophilia*	4	Gemmatales	4
.	.	TK10*	3	Gemmatimonadales*	4
.	.	Verrucomicrobiae	4	IMCC26256*	4
.	.	WS2_cl*	3	Lineage_IV	3
.	.	.	.	Micrococcales*	4
.	.	.	.	Micropepsales*	4
.	.	.	.	Nitrospirales*	3
.	.	.	.	Nostocales*	1
.	.	.	.	Obscuribacterales	4
.	.	.	.	Oligoflexales	4
.	.	.	.	Omnitrophicaeota_or	3
.	.	.	.	Oxyphotobacteria_Incertae_Sedis	1
.	.	.	.	Paracaedibacterales	1
.	.	.	.	Pedosphaerales	3
.	.	.	.	Phycisphaerales	3
.	.	.	.	Pla4_lineage_or*	3
.	.	.	.	RBG-13-54-9	3
.	.	.	.	RCP2-54*	4
.	.	.	.	Reyranellales*	4
.	.	.	.	Rhodobacterales*	1
.	.	.	.	Rubrobacterales*	3
.	.	.	.	S085*	3
.	.	.	.	Saccharimonadales*	4
.	.	.	.	Salinisphaerales*	4
.	.	.	.	SAR202_clade*	1
.	.	.	.	Selenomonadales*	4
.	.	.	.	Solibacterales*	4
.	.	.	.	Solirubrobacterales	4
.	.	.	.	Subgroup_17_or*	3
.	.	.	.	Subgroup_2*	4
.	.	.	.	Subgroup_25_or*	3
.	.	.	.	Subgroup_5_or	3
.	.	.	.	Subgroup_7	3
.	.	.	.	TK10_or	3
.	.	.	.	Unknown_Order	2
.	.	.	.	WD260*	4
.	.	.	.	WS2_or*	3

Table S19. Significant ($\alpha = 0.05$) family level ISA results for particular elevation levels (F = 1, WBS = 2, LS = 3, MBS = 4). Asterisked entries indicate significant (at $\alpha = 0.05$) indicators after adjustment for false discovery rate.

Family	lvl	Family	lvl	Family	lvl
A0839*	4	Hymenobacteraceae	2	Pla4_lineage_fa*	3
A21b*	4	IMCC26256_fa*	4	Planococcaceae*	4
Acidobacteriaceae_Subgroup_1*	4	Intrasporangiaceae*	4	Prolixibacteraceae*	4
Acidothermaceae*	4	Kineosporiaceae	3	Promicromonosporaceae*	2
AKYH767	3	Labraceae*	4	Pseudonocardiaceae	4
Alicyclobacillaceae*	1	Lineage_IV_fa	3	RBG-13-54-9_fa	3
Amoebophilaceae	4	Methylophilaceae*	4	RCP2-54_fa*	4
Anaerolineaceae*	3	Microbacteriaceae	4	Reyranellaceae*	4
Archangiaceae*	1	Microcystaceae*	1	Rhizobiales_Incertae_Sedis*	3
Armatimonadaceae*	2	Mycobacteriaceae*	4	Rhodanobacteraceae	4
Armatimonadales_fa	2	Nakamurellaceae*	4	Rhodobacteraceae*	1
Bacillaceae*	4	Nitrospiraceae*	3	Roseiflexaceae*	3
bacteriap25*	3	Obscuribacterales_fa	4	Rubrobacteriaceae*	3
BD2-11_terrestrial_group_fa	3	Paenibacillaceae*	4	Ruminococcaceae*	4
Burkholderiaceae*	4	Paracaedibacteraceae	1	S085_fa*	3
C0119_fa	3	Peptococcaceae*	4	Saccharimonadales_fa*	4
Caldilineaceae*	3	Phaselicystidaceae*	1	Saprospiraceae*	3
Candidatus_Peribacteria_fa	3	Phycisphaeraceae	3	SAR202_clade_fa*	1
CHAB-XI-27_fa*	1	Planococcaceae*	4	SC-I-84*	4
Chloroflexaceae	3	Promicromonosporaceae*	2	Solibacteraceae_Subgroup_3*	4
Chthoniobacteraceae*	4	RBG-13-54-9_fa	3	Solimonadaceae*	4
Chthonomonadales_fa*	3	Reyranellaceae*	4	Solirubrobacteraceae	4
Clostridiaceae_1*	4	Rhodanobacteraceae	4	Spirosomaceae*	1
Cryptosporangiaceae*	4	Roseiflexaceae*	3	Sporichthyaceae	4
Desulfarculaceae	4	Ruminococcaceae*	4	Subgroup_17_fa*	3
Diplorickettsiaceae*	4	Saccharimonadales_fa*	4	Subgroup_2_fa*	4
Entotheonellaceae*	3	SAR202_clade_fa*	1	Subgroup_25_fa*	3
Euzebyaceae	1	Solibacteraceae_Subgroup_3*	4	Subgroup_7_fa	3
FBP_fa*	1	Solirubrobacteraceae	4	Thermoactinomycetaceae*	4
Fibrobacteraceae*	3	Sporichthyaceae	4	TK10_fa	3
Frankiaceae*	4	Subgroup_2_fa*	4	Trueperaceae*	1
Gaiellaceae*	4	Subgroup_7_fa	3	Unknown_Family*	4
Gemmataceae	4	TK10_fa	3	Veillonellaceae*	4
Gemmatimonadaceae*	4	Unknown_Family*	4	WD260_fa*	4
Geobacteraceae*	4	WD260_fa*	4	WS2_fa*	3
Geodermatophilaceae*	4	Xanthobacteraceae*	4	Xanthobacteraceae*	4
.	.	.	.	Xiphinematobacteraceae*	4

Table S20. Significant ($\alpha = 0.05$) genus level ISA results for particular elevation levels (F = 1, WBS = 2, LS = 3, MBS = 4). Asterisked entries indicate significant (at $\alpha = 0.05$) indicators after adjustment for false discovery rate.

Genus	lvl	Genus	lvl	Genus	lvl
A0839_ge	4	Euzebya	1	Paucimonas	4
A21b_ge*	4	Flavisolibacter*	4	Pedobacter	4
Acetivibrio*	4	Gaiella*	4	Pedomicrobium*	4
Acidibacter*	4	Geobacter*	4	Pedosphaeraceae_ge	4
Acidipila*	4	Herbaspirillum*	4	Phaselicystis*	1
Acidothermus*	4	Hyphomicrobium*	4	Phreatobacter	4
Acidovorax*	1	Inquilinus*	4	Pla4_lineage_ge*	3
Actinocorallia	3	KD3-93_ge*	3	Planococcaceae_ge*	4
ADurb Bin063-1*	4	Kouleothrix*	3	Polaromonas	4
Agromyces*	2	Lautropia*	1	Polyangium	3
AKYH767_ge	3	Lentimicrobiaceae_ge*	4	Polycyclovorans*	3
alphaI_cluster	1	Leptothrix*	4	Porphyrobacter	1
Ammoniphilus*	4	Limnobacter*	4	Pseudarthrobacter*	4
Amycolatopsis*	4	Lineage_IV_ge*	3	Pseudonocardia	4
Anaeromyxobacter*	4	Methylocella*	4	RBG-13-54-9_ge	3
Archangium*	1	Microlunatus*	3	RCP2-54_ge*	4
Arenimonas	3	Microvirga*	1	Reyranella*	4
Armatimonadales_ge	2	Mucilaginibacter*	4	Rhizobacter*	4
Armatimonas*	2	Myxococcus*	1	Rhodococcus*	4
Arthrobacter*	4	Nitrobacter*	4	Rhodoferax	4
Bacillus*	4	Nitrosospira	4	Rhodopseudomonas	4
bacteriap25_ge*	3	Nordella	4	Rhodovarius	3
Bacteriovorax	4	Novosphingobium*	4	Roseiarcus*	4
Bryobacter*	4	Obscuribacterales_ge	4	Rubrobacter*	3
Burkholderia-Caballeronia-Paraburkholderia*	4	Oceanobacillus*	4	Ruminiclostridium_1*	4
C0119_ge	3	Ottowia*	4	S085_ge*	3
Candidatus_Adlerbacteria_ge	3	Pajaroellobacter*	4	Saccharimonadales_ge*	4
Candidatus_Amoebophilus	4	Paucibacter	4	Salinibacterium	4
Candidatus_Chlorothrix	3	Pedobacter	4	SAR202_clade_ge*	1
Candidatus_Entotheonella*	3	Pedosphaeraceae_ge	4	SC-I-84_ge*	4
Candidatus_Hydrogenedens	4	Phreatobacter	4	Sediminibacterium*	4
Candidatus_Koribacter*	4	Planococcaceae_ge*	4	Shimazuella	4
Candidatus_Peribacteria_ge	3	Polyangium	3	Solimonas*	4
Candidatus_Saccharimonas	4	Porphyrobacter	1	Solirubrobacter	4
Candidatus_Solibacter*	4	Pseudonocardia	4	Spirosoma*	1
Candidatus_Udaeobacter*	4	RCP2-54_ge*	4	Sporosarcina*	4
Candidatus_Xiphinematobacter*	4	Rhizobacter*	4	Stenotrophobacter*	4
Cephalothrix_SAG_75 79	1	Rhodoferax	4	Stenotrophomonas	3
CHAB-XI-27_ge*	1	Rhodovarius	3	Streptacidiphilus*	4
Chthonomonadales_ge	3	Rubrobacter*	3	Subgroup_17_ge*	3
Clostridioides*	4	S085_ge*	3	Subgroup_2_ge*	4
Clostridium_sensu_stricto*	4	Salinibacterium	4	Subgroup_25_ge*	3
Clostridium_sensu_stricto_10*	1	SC-I-84_ge*	4	Subgroup_5_ge	3
Cohnella*	4	Shimazuella	4	Subgroup_7_ge	3
Collimonas	4	Solirubrobacter	4	Thermoactinomyces*	4
Cryptosporangium*	4	Sporosarcina*	4	TK10_ge	3
Cystobacter*	1	Stenotrophomonas	3	Truepera*	1
Desulfosporosinus*	4	Subgroup_17_ge*	3	Tumebacillus*	1
Dinghuibacter*	4	Subgroup_25_ge*	3	Undibacterium*	1
Dokdonella*	4	Subgroup_7_ge	3	Urania-1B-19_marine_sediment_group	3
Domibacillus*	4	TK10_ge	3	Virgisporangium	3
Dysgonomonas	3	Tumebacillus*	1	Viridibacillus*	4
Ellin6067*	4	Urania-1B-19_marine_sediment_group	3	WCHB1-32*	4
Emticicia	3	Viridibacillus*	4	WD260_ge*	4
Endobacter*	3	WD260_ge*	4	WS2_ge*	3
Eoetvoesia*	4	Xylanimicrobium*	2	Xylanimicrobium*	2
.	.	.	.	Yonghaparkia*	4

5.2.2 Shrub

Table S21 provides richness and diversity summaries for OTUs and Linnean taxonomic summaries within shrub/intershrub levels. Table S22 summarizes significant ISA results across both shrub levels for each taxonomic hierarchy. Table S23 provides a summary of significant ISA results for each taxonomic hierarchy, for both shrub level. Table S24-S26 show indicator taxa, at various taxonomic level, understory shrubs or in intershrub spaces. Comparisons over broad patterns in phyla are also considered within the context of the NMDS ordination (Figure S4).

Table S21. Richness and diversity summary for shrub/intershrub levels. Except for β-diversity (which was measures as mean Bray-Curtis dissimilarity), entries are $\bar{x} \pm$ SEM.

	I	S
OTU richness	11325.06 ± 589.3	12837.12 ± 671.05
OTU $\alpha-$diversity	6.78 ± 0.12	7.08 ± 0.07
OTU $\beta-$diversity	NaN	0.53
No. Phylum	23.81 ± 0.21	24.88 ± 0.15
No. Class	65.81 ± 0.86	68.5 ± 0.74
No. Order	144.81 ± 2.14	152.75 ± 2.04
No. Family	232.38 ± 4.3	247.69 ± 3.51
No. Genus	371.44 ± 8.29	398.81 ± 6.99

Table S22. Overall significant ($\alpha = 0.05$) ISA results for shrub/intershrub levels for the indicated taxonomic hierarchies.

	Phylum	Class	Order	Family	Genus
No. of taxa considered	24	61	146	247	410
No. unadj. signif. indicators	10	23	56	78	99
No. FDR-adj. signif. indicators	6	12	28	40	42
Prop. of unadj. signif. indicators	0.4167	0.377	0.3836	0.3158	0.2415
Prop. signif. FDR-adj. indicators	0.25	0.1967	0.1918	0.1619	0.1024

Table S23. Numbers of significant ($\alpha = 0.05$) ISA results for shrub/intershrub levels for the indicated taxonomic hierarchies. Semicolons separate significant unadjusted and FDR-adjusted results.

	Intershrub	Shrub
Phylum	1;1	9;5
Class	2;1	21;11
Order	3;1	53;27
Family	4;1	74;39
Genus	4;2	95;40

Table S24. Significant ($\alpha = 0.05$) ISA at the phylum, class, and order level for particular shrub/intershrub levels (Intershrub = 1, Shrub = 2). Asterisked entries indicate significant (at $\alpha = 0.05$) indicators after adjustment for false discovery rate.

Phylum	lvl	Class	lvl	Order	lvl
Acidobacteria	2	ABY1*	2	Armatimonadales	1
Chlamydiae*	2	Acidimicrobiia	2	Babeliales*	2
Deinococcus-Thermus*	1	Alphaproteobacteria	2	Bacteroidales	2
Dependentiae*	2	Armatimonadia	1	Bdellovibrionales	2
Elusimicrobia	2	Babeliae*	2	Candidatus_Adlerbacteria*	2
Hydrogenedentes*	2	Chlamydiae*	2	Candidatus_Amesbacteria	2
Omnitrophicaeota*	2	Deinococci*	1	Candidatus_Kaiserbacteria	2
Patescibacteria*	2	Deltaproteobacteria	2	Candidatus_Moranbacteria*	2
Planctomycetes	2	Elusimicrobia	2	Candidatus_Pacebacteria*	2
Proteobacteria	2	Gracilibacteria	2	Candidatus_Peregrinibacteria	2
.	.	Hydrogenedentia*	2	Candidatus_Peribacteria	2
.	.	Ignavibacteria*	2	Candidatus_Uhrbacteria*	2
.	.	Melainabacteria*	2	Caulobacterales*	2
.	.	Microgenomatia*	2	CCD24*	2
.	.	OM190*	2	Chlamydiales*	2
.	.	Omnitrophicaeota_cl*	2	Corynebacteriales	2
.	.	Parcubacteria*	2	Deinococcales*	1
.	.	Planctomycetacia	2	Diplorickettsiales	2
.	.	S0134_terrestrial_group	2	Flavobacteriales	2
.	.	Saccharimonadia	2	Gammaproteobacteria_Incertae_Sedis*	2
.	.	Subgroup_17	2	Hydrogenedentiales*	2
.	.	Subgroup_5	2	Legionellales*	2
.	.	Subgroup_6*	2	Lineage_IV	2
.	.	.	.	Micavibrionales*	2
.	.	.	.	Micropepsales	2
.	.	.	.	Microtrichales*	2
.	.	.	.	Myxococcales	2
.	.	.	.	Nostocales	1
.	.	.	.	Obscuribacterales*	2
.	.	.	.	OM190_or*	2
.	.	.	.	Omnitrophicaeota_or*	2
.	.	.	.	OPB56	2
.	.	.	.	Parcubacteria_or*	2
.	.	.	.	Pirellulales	2
.	.	.	.	Planctomycetales*	2
.	.	.	.	Pseudomonadales	2
.	.	.	.	Pseudonocardiales	2
.	.	.	.	Reyranellales*	2
.	.	.	.	Rhizobiales	2
.	.	.	.	Rhodothermales	2
.	.	.	.	Rickettsiales*	2
.	.	.	.	S0134_terrestrial_group_or	2
.	.	.	.	Saccharimonadales	2
.	.	.	.	SBR1031*	2
.	.	.	.	SJA-28*	2
.	.	.	.	Solirubrobacterales	2
.	.	.	.	Sphingobacteriales*	2
.	.	.	.	Sphingomonadales	2
.	.	.	.	Steroidobacterales*	2
.	.	.	.	Streptosporangiales	2
.	.	.	.	Subgroup_17_or	2
.	.	.	.	Subgroup_5_or*	2
.	.	.	.	Subgroup_6_or*	2
.	.	.	.	Tepidisphaerales	2
.	.	.	.	Verrucomicrobiales*	2
.	.	.	.	Xanthomonadales	2

Table S25. Significant ($\alpha = 0.05$) family level ISA results for shrub/intershrub levels (Intershrub = 1, Shrub = 2). Asterisked entries indicate significant (at $\alpha = 0.05$) indicators after adjustment for false discovery rate.

Family	lvl	Family	lvl	Family	lvl
A0839	2	Lineage_IV_fa	2	Rubinisphaeraceae*	2
A4b*	2	Micavibrionaceae*	2	Rubritaleaceae	2
AKYH767*	2	Microscillaceae*	2	S0134_terrestrial_group_fa	2
Bdellovibrionaceae	2	Nannocystaceae	2	Saccharimonadaceae*	2
Candidatus_Adlerbacteria_fa*	2	NS9_marine_group*	2	Saccharimonadales_fa	2
Candidatus_Amesbacteria_fa	2	Oligoflexaceae	2	Sandaracinaceae*	2
Candidatus_Kaiserbacteria_fa	2	Omnitrophicaeota_fa*	2	Saprospiraceae	2
Candidatus_Moranbacteria_fa*	2	P3OB-42	2	SBR1031_fa	1
Candidatus_Pacebacteria_fa	2	Parcubacteria_fa*	2	Schlesneriaceae*	2
Candidatus_Peregrinibacteria_fa	2	Pirellulaceae	2	SJA-28_fa*	2
Candidatus_Peribacteria_fa	2	Pseudonocardiaceae	2	SM2D12*	2
Candidatus_Uhrbacteria_fa*	2	Rhizobiaceae*	2	Solirubrobacteraceae	2
Caulobacteraceae*	2	Rhodothermaceae	2	Sphingobacteriaceae	2
CCD24_fa*	2	Rubritaleaceae	2	Sphingomonadaceae	2
Cryptosporangiaceae	2	Saccharimonadaceae*	2	Steroidobacteraceae*	2
Deinococcaceae*	1	Sandaracinaceae*	2	Streptosporangiaceae*	2
Devosiaceae*	2	SBR1031_fa	1	Subgroup_17_fa	2
Diplorickettsiaceae	2	SJA-28_fa*	2	Subgroup_5_fa	2
env OPS_17*	2	Solirubrobacteraceae	2	Subgroup_6_fa*	2
Flavobacteriaceae*	2	Sphingomonadaceae	2	Terrimicrobiaceae	2
Haliangiaceae*	2	Streptosporangiaceae*	2	TRA3-20*	2
Hydrogenedensaceae*	2	Subgroup_5_fa	2	Unknown_Family	2
Iamiaceae	2	Terrimicrobiaceae	2	Verrucomicrobiaceae*	2
Ilumatobacteraceae*	2	Unknown_Family	2	VHS-B4-70*	2
Inquilinaceae	2	VHS-B4-70*	2	WD2101_soil_group	2
KD3-93	2	Xanthobacteraceae	2	Xanthobacteraceae	2

Table S26. Significant ($\alpha = 0.05$) genus level ISA results for shrub/intershrub levels (Intershrub = 1, Shrub = 2). Asterisked entries indicate significant (at $\alpha = 0.05$) indicators after adjustment for false discovery rate.

Genus	lvl	Genus	lvl	Genus	lvl
A0839_ge	2	Flavobacterium*	2	Phenylobacterium	2
A21b_ge	2	Gemmata	2	Phyllobacterium	2
A4b_ge*	2	Iamia	2	Pigmentiphaga	2
Acidibacter	2	Jahnella	2	Planctopirus*	2
Acidipila	2	Legionella*	2	Pseudomonas	2
AKYH767_ge	2	Loktanella	1	Reyranella*	2
Allo-Neo-Para-rhizobium	2	Luteolibacter	2	Rhizobacter	2
Aminobacter	2	Mesorhizobium*	2	Rhodanobacter*	2
Arenimonas	2	Microbacterium*	2	Rhodoferax*	2
Bdellovibrio	2	Myxococcus	1	Rhodopseudomonas*	2
Bosea*	2	Nocardioides	2	Roseimaritima	2
Brevundimonas*	2	NS11-12_marine_group_ge*	2	S0134_terrestrial_group_ge	2
Candidatus_Adlerbacteria_ge*	2	Obscuribacterales_ge*	2	Saccharimonadales_ge	2
Candidatus_Amesbacteria_ge	2	OM190_ge*	2	Sandaracinus*	2
Candidatus_Kaiserbacteria_ge	2	OPB56_ge	2	Sediminibacterium*	2
Candidatus_Moranbacteria_ge*	2	Paradevosia	2	SH-PL14*	2
Candidatus_Pacebacteria_ge*	2	Phenylobacterium	2	SJA-28_ge*	2
Candidatus_Peregrinibacteria_ge	2	Pigmentiphaga	2	SM2D12_ge*	2
Candidatus_Peribacteria_ge	2	Pseudomonas	2	Solirubrobacter	2
Candidatus_Protochlamydia*	2	Rhizobacter	2	Sphingobium	2
Candidatus_Saccharimonas*	2	Rhodoferax*	2	Sphingomonas	2
Candidatus_Uhrbacteria_ge*	2	Roseimaritima	2	Sporosarcina*	1
Caulobacter*	2	Saccharimonadales_ge	2	Stenotrophobacter	2
CCD24_ge	2	Sediminibacterium*	2	Steroidobacter*	2
CL500-29_marine_group*	2	SJA-28_ge*	2	Streptosporangium*	2
Clavibacter*	2	Solirubrobacter	2	Subgroup_17_ge	2
Cryptosporangium	2	Sphingomonas	2	Subgroup_5_ge	2
Dactylosporangium	2	Stenotrophobacter	2	Subgroup_6_ge*	2
Deinococcus*	1	Streptosporangium*	2	Taibaiella*	2
Devosia*	2	Subgroup_5_ge	2	TRA3-20_ge	2
Dokdonella	2	Taibaiella*	2	Variovorax*	2
Dyadobacter	2	Variovorax*	2	VHS-B4-70_ge*	2
env OPS_17_ge*	2	WD2101_soil_group_ge	2	WD2101_soil_group_ge	2

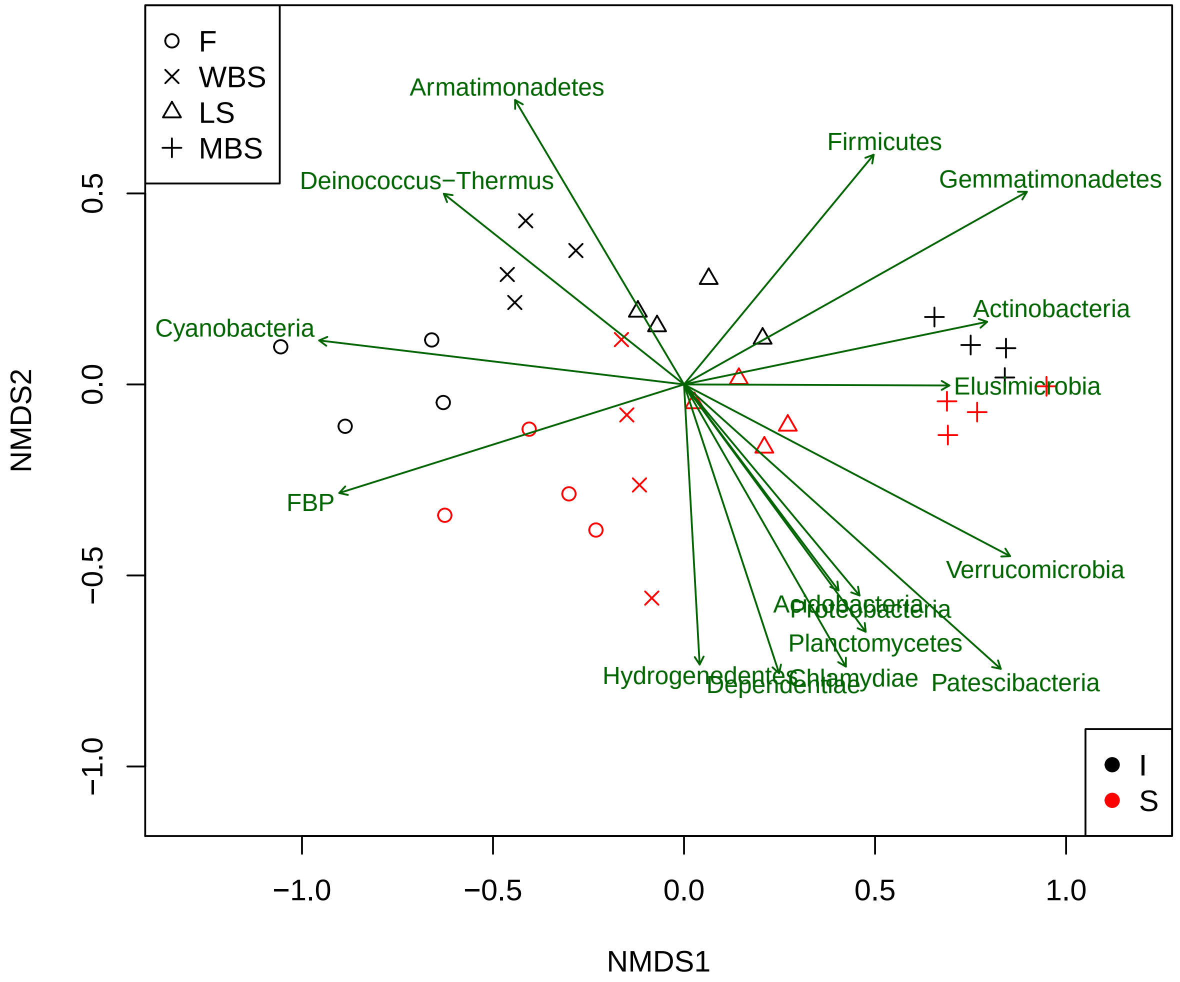

Figure S4. NMDS ordination shown in Figure S3, with biocrust phylum distribution patterns overlain. Only significant phylum correlates (p-values ≤ 0.05) are shown.

5.3 Nitrogen-cycling Traits Analysis

We used PICRUSt to estimate biocrust nitrogen traits. For each biocrust sample, the relative abundance of a specific gene was calculated as copies of that gene per average bacterial genome after considering 16S rRNA gene copies per genome. Our estimation shows a linear decrease in the relative abundance of nitrogenase genes *nifD* (K02586), *nifK* (K02591), and *nifH* (K02588) and a linear increase in the relative abundance of *anfG* (K00531) along the elevation gradient (Figure S5). Because *nifHDK* dominated among all nitrogenase genes in the studied BSC communities, the overall relative abundance of nitrogenase genes decreased along the climatic elevation gradient.

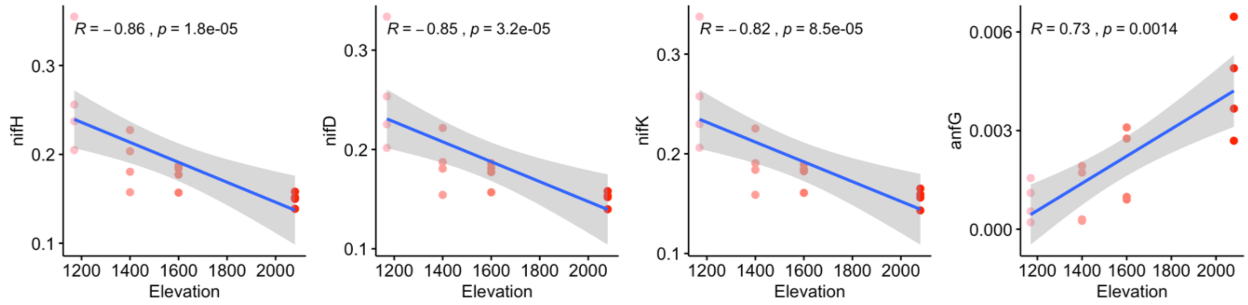

Figure S5. Relative abundance of nitrogenase genes along the elevational gradient. Also shown are Spearman's rank correlation results, 95% confidence interval, and linear regression lines.

References

Aho, K. (2018) *asbio: A Collection of Statistical Tools for Biologists*. R package version 1.5-3.

Aho, K.A. (2016) *Foundational and Applied Statistics for Biologists using R*. Chapman and Hall/CRC.

Bates, D., Mächler, M., Bolker, B. & Walker, S. (2015) Fitting linear mixed-effects models using lme4. *Journal of Statistical Software*, **67**, 1–48.

Bray, J.R. & Curtis, J.T. (1957) An ordination of the upland forest communities of southern Wisconsin. *Ecological monographs*, **27**, 325–349.

Dufrene, M. & Legendre, P. (1997) Species assemblages and indicator species: the need for a flexible asymmetrical approach. *Ecological monographs*, **67**, 345–366.

Kuznetsova, A., Brockhoff, P.B. & Christensen, R.H.B. (2017) lmerTest package: Tests in linear mixed effects models. *Journal of Statistical Software*, **82**, 1–26.

Legendre, P. & Legendre, L.F. (2012) *Numerical Ecology*, volume 24. Elsevier.

Oksanen, J., Blanchet, F.G., Friendly, M., Kindt, R., Legendre, P., McGlinn, D., Minchin, P.R., O'Hara, R.B., Simpson, G.L., Solymos, P., Stevens, M.H.H., Szoecs, E. & Wagner, H. (2018) *vegan: Community Ecology Package*. R package version 2.5-2.

R Core Team (2018) *R: A Language and Environment for Statistical Computing*. R Foundation for Statistical Computing, Vienna, Austria.

Roberts, D.W. (2016) *labdsv: Ordination and Multivariate Analysis for Ecology*. R package version 1.8-0.
